# A procession of metabolic alterations accompanying muscle senescence in ***Manduca sexta***

**DOI:** 10.1038/s41598-018-19630-5

**Published:** 2018-01-17

**Authors:** Bernard W. M. Wone, Jason M. Kinchen, Elana R. Kaup, Beate Wone

**Affiliations:** 1 Department of Biology, University of South Dakota, Vermillion, SD 57069 USA; 2 Metabolon Inc., Durham, NC 27713 USA

## Abstract

Biological aging profoundly impairs muscle function, performance, and metabolism. Because the progression of metabolic alterations associated with aging muscle has not been chronicled, we tracked the metabolic profiles of flight muscle from middle to advanced age in *Manduca sexta* to identify key molecules during the progression of muscle aging, as well as to evaluate the utility of the *M. sexta* system for molecular dissection of muscle aging. We identified a number of differences between Diel Time, Sexes, and Muscle Ages, including changes in metabolites related to energetics, extracellular matrix turnover, and glutathione metabolism. Increased abundances of glycolytic metabolites suggest a shift toward increased glycolysis with advancing age, whereas decreased abundances in lysolipids and acylcarnitines reflect decreasing *beta*-oxidation. We also observed a shift towards decreased polyamine metabolism with age, which might result in an age-related decline in lipid metabolism possibly due to regulation of energy metabolism by polyamines. Collectively, our findings demonstrate the feasibility of our system and approach and provide a deeper understanding of lepidopteran aging. More importantly, the results identify the key altered metabolic pathways that collectively contribute to the muscle aging phenotype and thereby improve our understanding of muscle senescence.

## Introduction

Muscle aging is a complex, multifactorial process^1–4^ that inevitably results in the dysfunction, reduced performance, and reduced mass of muscles^5,6^. Not surprisingly, the mechanistic basis of muscle senescence has not only attracted the interest of medical biologists and human physiologists, but also of ecologists^7–15^, and evolutionary biologists^16,17^ as an important problem in biology^4^.

The mechanisms underlying muscle aging are likely to involve many different pathways as well as interactions among them. Prior to the emergence of high-throughput technologies, research into the mechanistic basis of muscle aging has concentrated largely on a specific single level of organization and has focused on individual components of the process in isolation (one gene or protein at a time) or static time points (only young and old age classes). But recently, a more multi-dimensional scale ‘omics approach has emerged^18,19^.

We are just beginning to understand the scope of the molecular mechanisms of muscle aging^20,21^. Transcriptional profiling of 81 human muscle biopsy samples has identified a 250-gene signature for muscle aging^20^. Compared to age-associated gene regulation in other tissues, these authors found increased expression of pathways regulating cell growth, complement activation, and ribosomal and extracellular matrix genes, but decreased expression of genes for chloride transport and mitochondrial oxidative phosphorylation during muscle aging. Similarly, a meta-analysis of microarray data comparing old and young mice, rats, and humans across a variety of tissues revealed that 73 genes had altered expression upon aging, with increased expression of genes involved in inflammation and immune responses, and reduced expression of genes essential to energy metabolism, especially mitochondrial genes^22^. Strikingly, the mammalian target of the rapamycin (mTOR) signaling pathway is thought to regulate mitochondrial function^23^. Specifically, down-regulation of the mTOR pathway, including mTOR complex I (mTORC1) altered metabolism^24–27^ and was associated with a decline in muscle function. Collectively, these studies suggest that mitochondrial dysfunction appears to have a key role in age-related decline of muscle function^28^. Indeed, a more recent meta-study reported 957 genes associated with perturbation of many central metabolic pathways, including reduced expression of mitochondrial genes such as those encoding ATP synthase, NADH dehydrogenase, cytochrome C reductase, oxidase complexes, as well as enzymes involved in glucose and pyruvate processing^21^.

Age-related changes in gene expression in muscle are likely correlated with the dynamics in metabolite levels. Hence, metabolomics approaches can provide a detailed understanding of organismal phenotypes^29,30^ and responses to advancing age^31^. Indeed, recent studies reported that aging muscles showed perturbations in lipid and glucose metabolism in mice^32^ and in rats^33^, which is consistent with the mitochondrial dysfunction observed in aging muscles^32,33^. Furthermore, Fazelzadeh *et al*.^34^ reported that the primary differences in skeletal muscle metabolite levels between healthy young and older humans were related to mitochondrial function, muscle fiber type, and tissue turnover. The limitations of these metabolomics studies, however, lie in the fact that global metabolites were only assayed at single time points in young and older individuals and thus failed to capture and track true age-related phenomena during the muscle aging process^35–37^. We therefore aimed to obtain a time course profile of metabolites in senescing muscles to elucidate the key molecules regulating or regulated by the progression of muscle senescence that leads to decreased muscle performance and function at advanced age in *M. sexta*.

Here we report a time series analysis of the flight muscle metabolome of *Manduca sexta* at middle to advanced age to characterize the concerted biochemical and molecular changes that occur during muscle aging. *Manduca sexta* was used as a model for several reasons. First, colonies of *M. sexta* can be maintained at reasonable cost. Second, the genome of *M. sexta* has been sequenced. Third, adult *M. sexta* has a short lifespan, which enables us to characterize the entire muscle aging process across time. Using this approach, we can identify and track metabolic changes that can then be used as age-related biomarkers of muscle dysfunction during muscle senescence. Our results demonstrate the feasibility of the metabolomics approach and provide a deeper understanding of lepidopteran aging in general, and more importantly, a more comprehensive phenotype of muscle senescence.

## Results

### Lifespan Estimation

**Figure 1:**
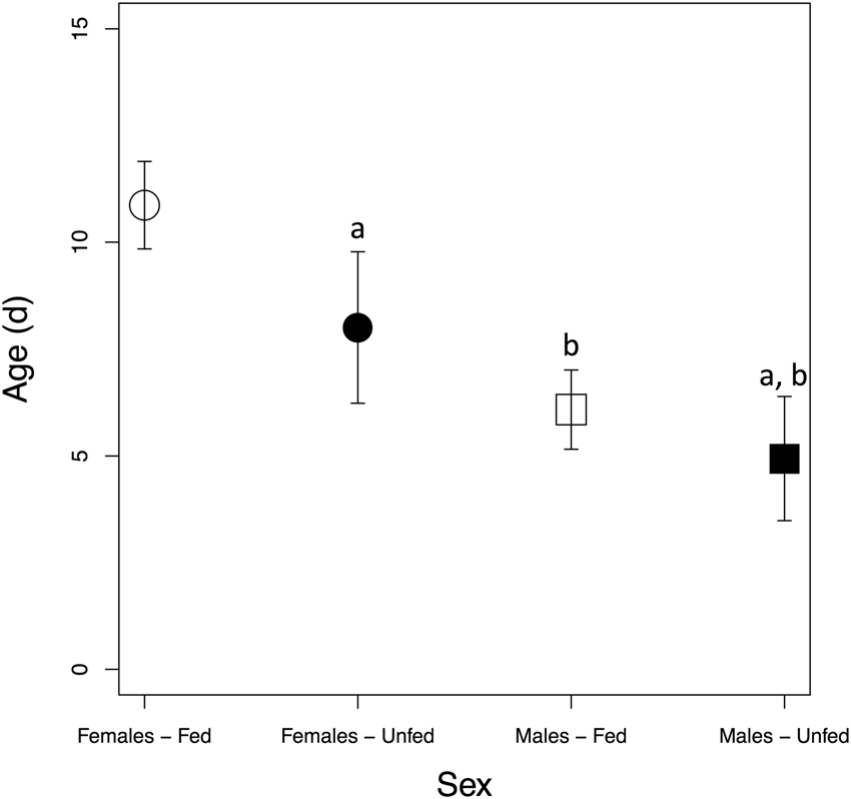
Lifespan of fed and unfed adult *Manduca sexta*. Error bars represent standard error of mean lifespan. Lowercase letters indicate significant pairwise comparisons: (**a**) Adjusted *P* < 0.05 compared to fed females; and (**b**) adjusted *P* < 0.001 compared to fed females. d = days.

Overall, Sex had a significant effect on lifespan (*t* = −6.94, *P* < 0.001) in *M. sexta*, as did feeding treatment (*t* = −2.80, *P* = 0.006). Females had a significantly longer lifespan compared to males (Fig. 1). *Ad lib* feeding extended the lifespan of females (fed females (*n* = 30) lifespan 10.9 days, range 5–17 d; unfed females (*n* = 10) lifespan 8.0 d, range 6–10 d; adjusted *P* = 0.03), but not of males (fed males (*n* = 37) lifespan 6.1 days, range 3–12 d; unfed males (*n* = 15) 4.9 days, range 2–8 d; *P* = 0.53).

### Metabolome Composition of *M. sexta* Flight Muscle

A total of 535 metabolites were characterized across Diel Time from middle to advanced Age to track patterns of metabolite abundance changes during muscle senescence in *M. sexta*. The detected metabolites were mapped onto general biochemical pathways and categorized into classes by prevalence for lipids (44%), followed by amino acids (24%), nucleotides (8%), carbohydrates (7%), cofactors (7%), and energy-related metabolites (2%), the latter of which were the least prevalent (Fig. S1a). Metabolic pathways with greater than twofold significant enrichment include those for biotin (8.23-fold), fatty acids (8.23-fold), phosphatidylserine (8.23-fold), guanine-containing purines (8.23-fold), polyamines (4.94-fold), branched chain amino acids (4.12-fold), benzoate (3.53-fold), TCA cycle metabolites (3.09-fold), sphingolipids (2.84-fold), nucleotide sugars (2.74-fold), fatty acyl carnitine (2.29-fold), ascorbate and aldarate (2.06-fold), glycerolipids (2.06-fold), monoacylglycerol (2.06-fold), and metabolites involved in vitamin B6 (2.06-fold) synthesis or function (Fig. S1b).

### Metabolic Differences Across Age, Diel Time, and Sex

**Figure 2:**
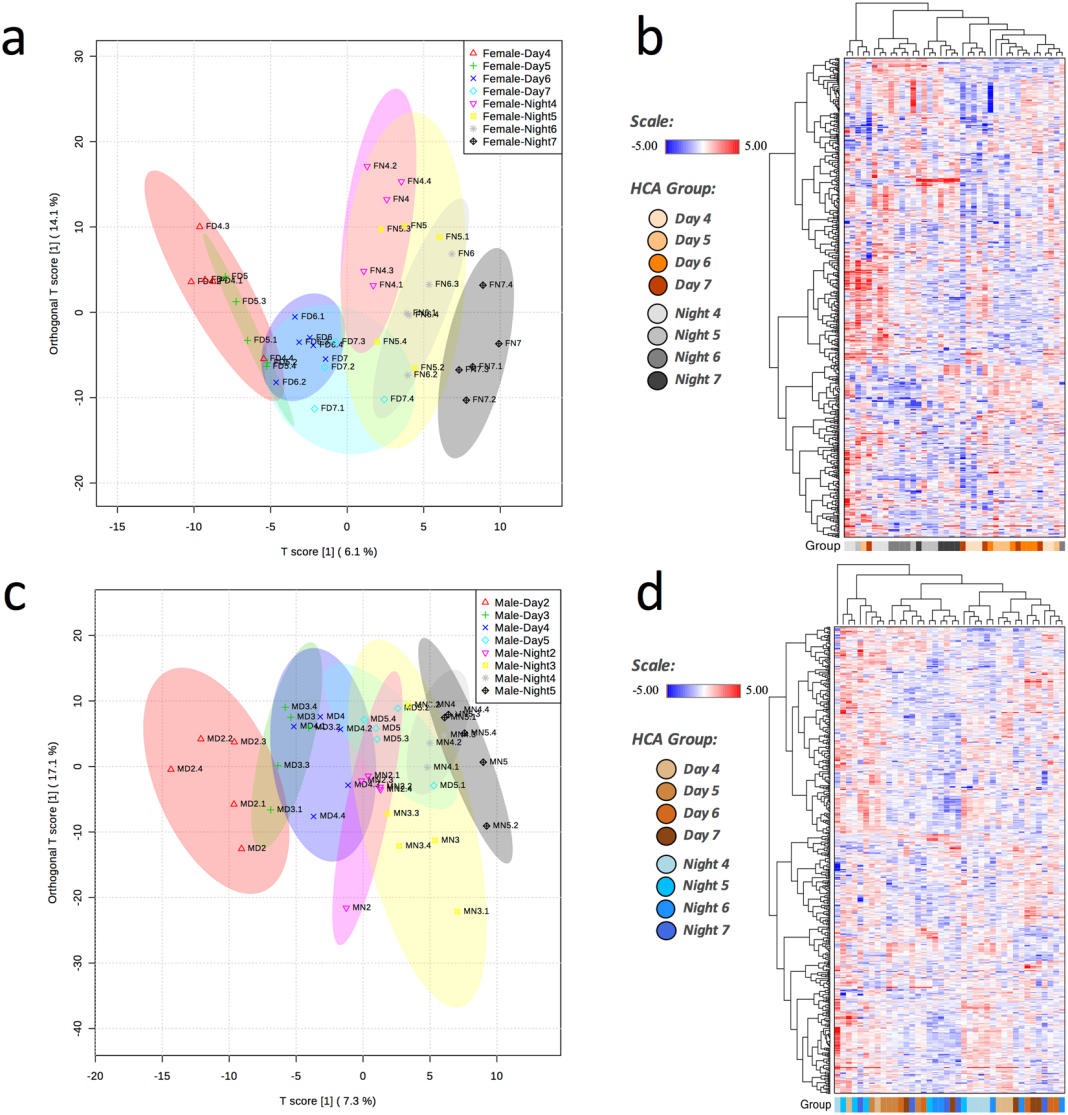
Metabolite distribution in flight muscle of senescing *Manduca sexta*, as determined by Orthogonal Partial Least Squares Discriminant Analysis (OPLS-DA), and as clustered by Hierarchical Cluster Analysis (HCA). Female: (**a)** OPLS-DA ordination (ellipses represent 95% confidence regions); (**b)** HCA. Male: (**c)** OPLS-DA ordination (ellipses represent 95% confidence regions); (**d)** HCA.

Orthogonal Partial Least Squares Discriminant Analysis (OPLS-DA) was used to analyze between-group variation and to provide a high-level overview of the dataset. In addition, Hierarchical Cluster Analysis (HCA) was used to assess sample similarity. Initial Principle Components Analysis (PCA) modeling separated samples by Sex (results not shown); thus, separate OPLS-DA and HCA for each Sex are presented. Female moth samples could be clearly distinguished according to metabolite abundance depending on Day (D) or Night (N) sampling and Age (model *Q*^2^ = 0.75, *p* < 0.001 after 1,000 permutations and *R*^2^*Y* = 0.94, *p* < 0.001 after 1,000 permutations, Fig. 2a). This result suggests circadian regulation of these differences and aging-associated changes in female muscle. As expected, female moths showed considerable within-group variation along the *y*-axis (groups are not tightly clustered along the *y*-axis, Fig. 2a), which indicates that individuals age differently. In the HCA, samples clustered into populations according to Diel Time (Day or Night) consistent with observations from the OPLS-DA (Fig. 2b). Similar to female samples, the OPLS-DA separated male samples based on Diel Time (Day or Night) and Age along the *x*-axis (model *Q*^2^ = 0.52, *P* < 0.001 after 1,000 permutations and *R*^2^*Y* = 0.88, *P* < 0.04 after 1,000 permutations, Fig. 2c), but showed less separation of advanced Age males according to Diel Time (Day or Night). Samples tended to show moderate clustering by group, with Day and Night samples clustering together (Fig. 2d). Early samples also tended to sort to proximal branches, with several exceptions.

### Biomarkers of Muscle Aging

Clear differences in muscle metabolite levels across Diel Time and Age prompted us to identify biomarkers for muscle aging using Random Forest Analysis (RFA), a statistical tool that bases supervised classification on an ensemble of decision trees. RFA assesses samples by Sex and Diel Time and shows variable predictive accuracy, although its predictive accuracy is above that expected by random chance, 25% for all RFA runs (Fig. S2). However, RFA highlighted biochemicals in similar metabolite classes, such as carbohydrate (ribose 5-phosphate, lactate, and maltose) or lipid (coenzyme A, sphingolipids, acylcarnitines, and fatty acids), as well as those related to arginine metabolism (5-methylthioadenosine, spermidine). A modified amino acid that is typically derived from collagen turnover, 5-hydroxylysine, was also highlighted in the RFA for each sex, which could reflect differences in muscle regeneration or ultrastructure associated with aging (discussed further below).

### A Procession of Metabolic Changes in Senescing Muscle

**Figure 3:**
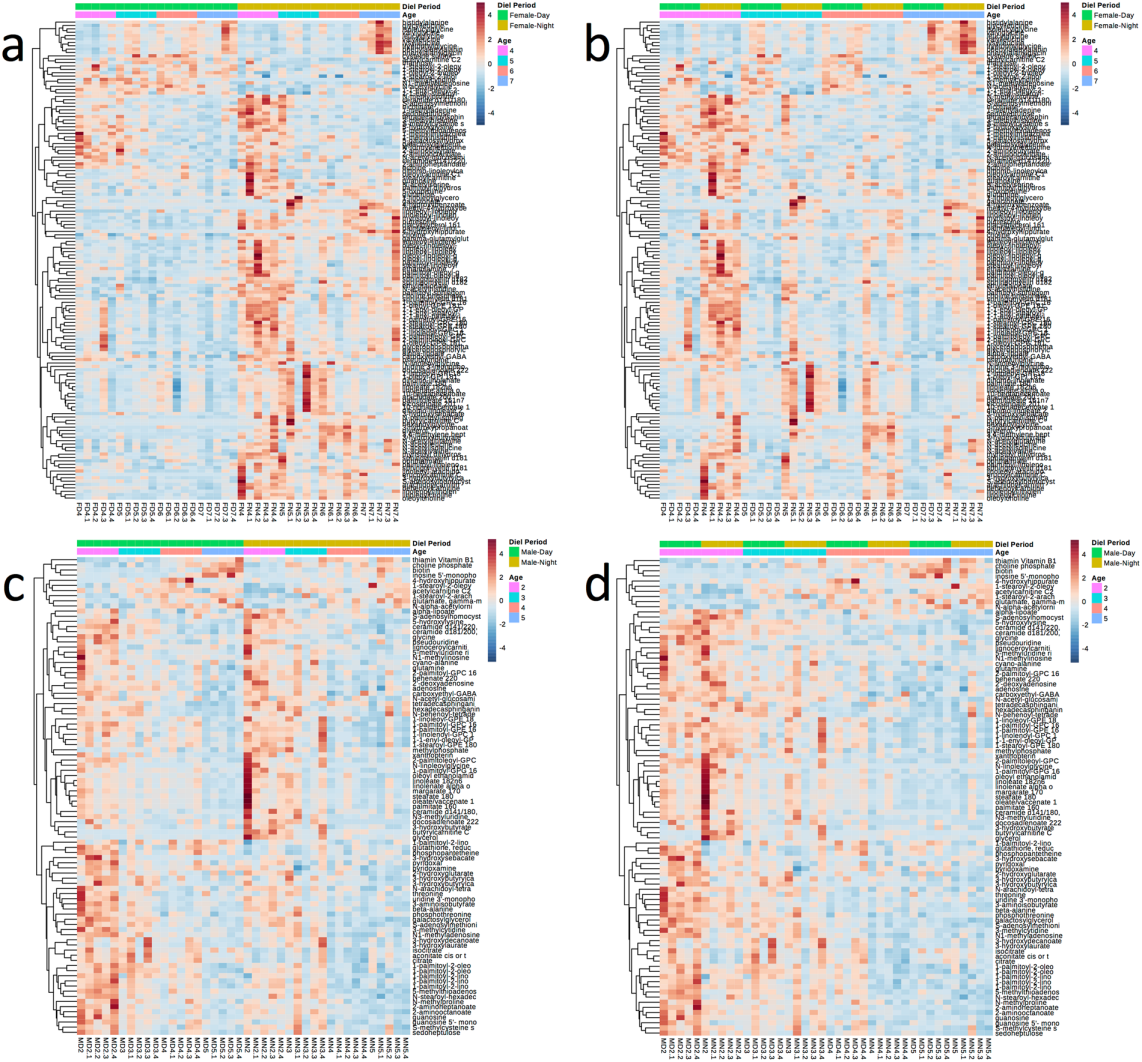
Metabolite abundances in muscle of *Manduca sexta* and their changes across Diel Time and Age. Female: (**a**) clustered by Diel Time; (**b**) clustered by Age. Male: (**c**) clustered by Diel Time; (**d**) clustered by Age. The adjusted *P* values for the comparisons are in Tables S1 and S2. Color in the heatmaps reflects the relative metabolite abundance: red represents metabolite abundances higher than the mean and blue represents metabolite abundances lower than the mean.

Significant changes were detected in the metabolic profiles of females and males, revealing both metabolite similarities and differences across Diel Time and Age. Interestingly, 108 metabolites showed diel oscillations (*P* adjusted < 0.05, Fig. 3a, Table S1) in abundance in females, and this pattern attenuated as females aged (Fig. 3b). In addition, the abundance of 50 metabolites significantly changed (*P* adjusted < 0.05) with Age in females (Fig. 3b, Table S1). Although males showed much reduced diel oscillations (17 metabolites, Fig. 3c), the abundance of 85 metabolites significantly changed (*P* adjusted < 0.05, Fig. 3d) with Age in males (Table S2).

### Changes in Collagen-associated Metabolites

**Figure 4:**
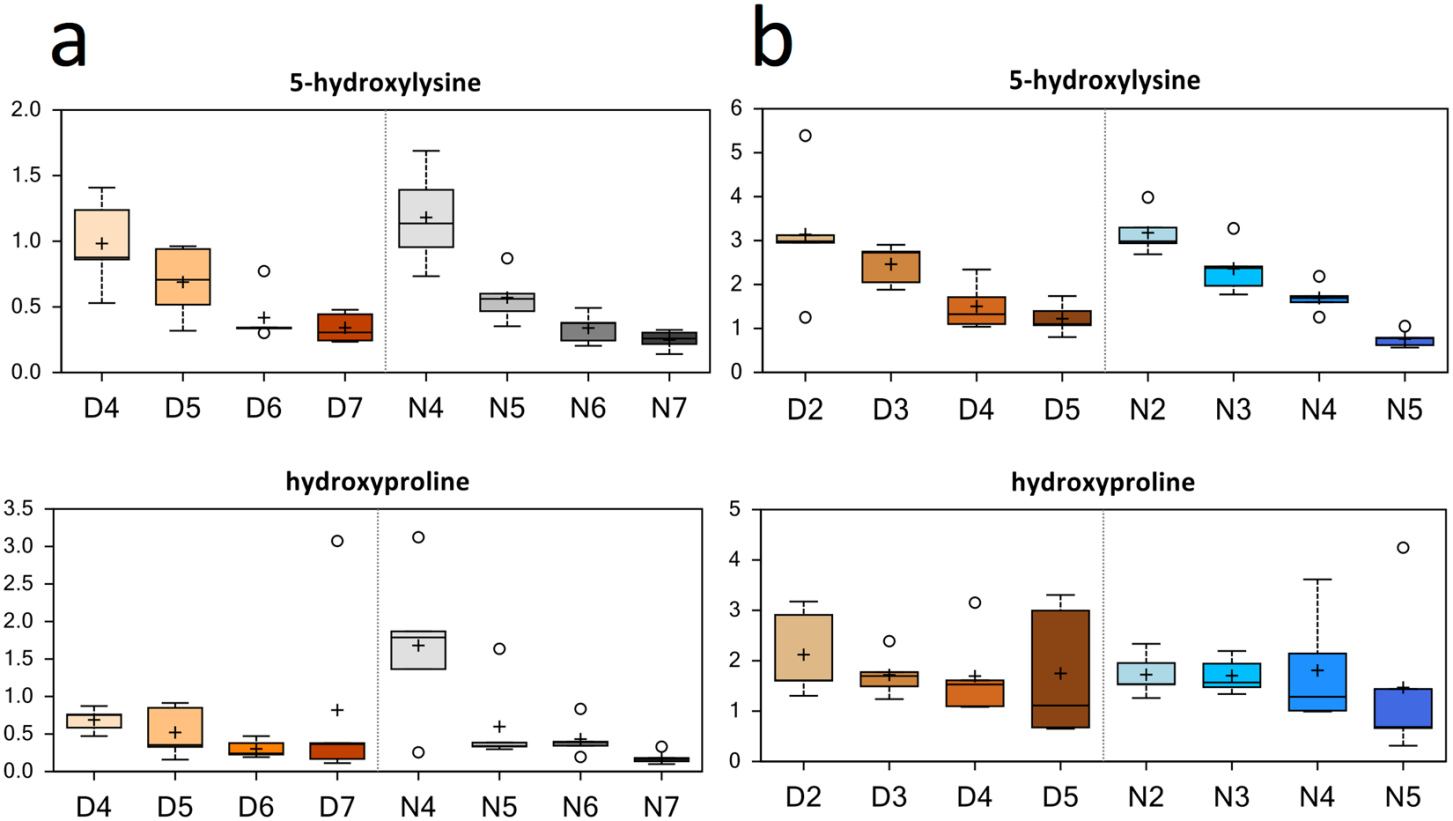
Differences in the abundances of modified amino acids derived from collagen turnover in muscle of *Manduca sexta* across Diel Time and Age. (**a**) Female, (**b**) Male. The *x*-axis represents Age post-eclosion at Day (D) and Night (N). The *y*-axis box plots indicate the scaled intensity mean (+) and median (−) values: upper and lower ranges of boxes indicate upper and lower quartiles, respectively; upper and lower whiskers indicate the maximum and minimum distributions of the data; small circles represent outlying data points. The adjusted *P* values for the significant comparisons are presented in Tables S1 and S2.

The extracellular matrix in muscle serves a crucial role in the transmission of force and maintenance of muscle elasticity. A modified amino acid derived from collagen turnover, 5-hydroxylysine, decreased in females (compare D5 to D4, D6 to D5, N5 to N4, and N6 to N5, Fig. 4a) and in males (compare D4 to D3, and N5 to N4, Fig. 4b). *Trans*-4-hydroxyproline, another modified amino acid derived from collagen, also decreased in females (compare N5 to N4, and N7 to N6), suggesting changes in collagen turnover or remodeling, or possibly changes in protein hydroxylation, in aging muscle.

### Decrease in Polyamine Metabolism

**Figure 5:**
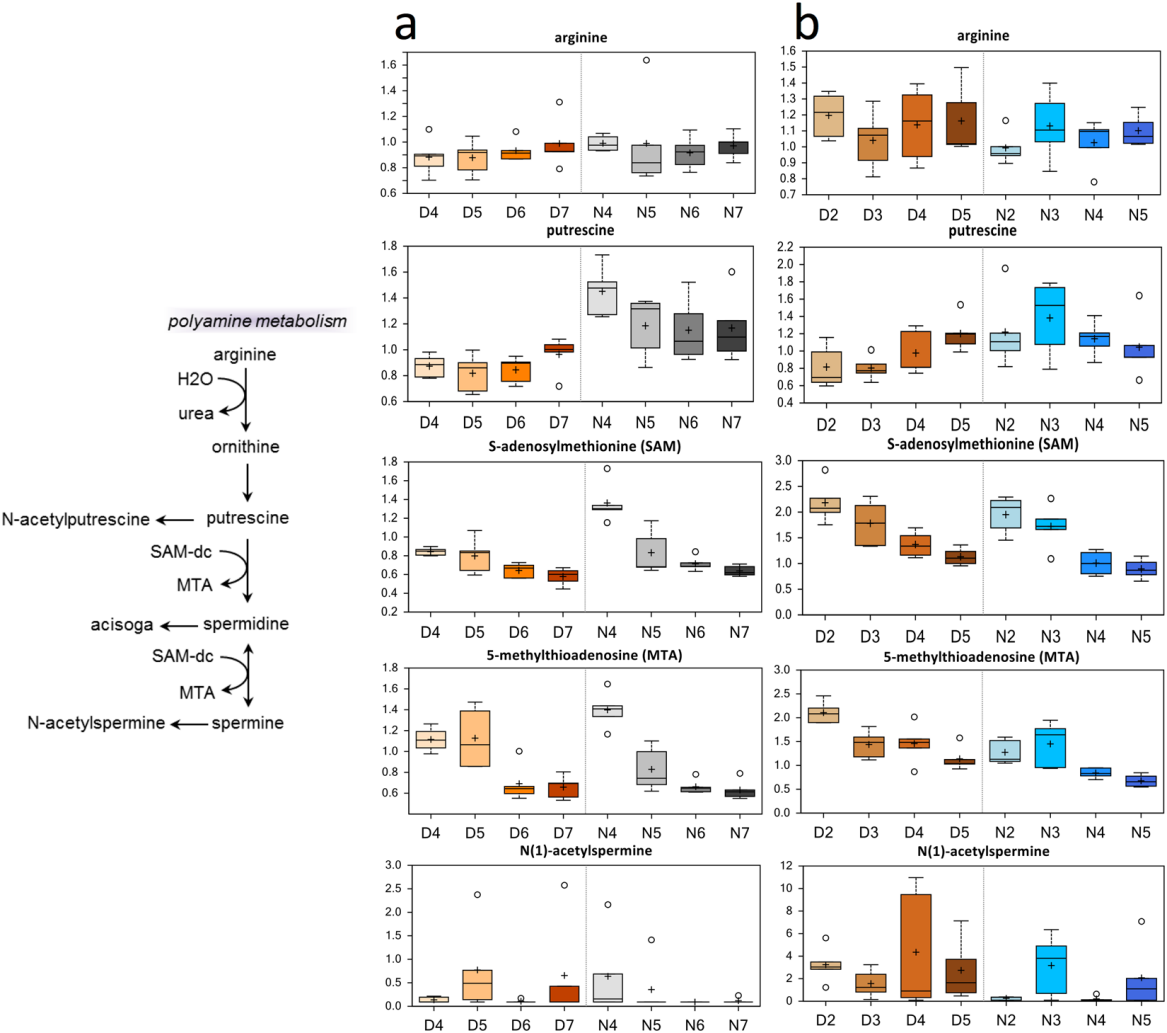
Differences in polyamine abundances in flight muscle of *Manduca sexta* across Diel Time and Age. (**a**) Female, (**b**) Male. The *x*-axis represents age post-eclosion at Day (D) and Night (N). The *y*-axis box plots indicate the scaled intensity mean (+) and median (−) values: upper and lower ranges of boxes indicate upper and lower quartiles, respectively; upper and lower whiskers indicate the maximum and minimum distributions of the data; small circles represent outlying data points. The adjusted *P* values for the significant comparisons are presented in Tables S1 and S2.

Significant decreases in the abundances of S-adenosylmethionine (SAMe) and 5-methylthioadenosine (MTA) were observed in both advanced Age females and males. SAMe is decarboxylated to SAM-dc, which is then converted to MTA (Fig. 5). Although changes in N(1)-acetylspermin**e** levels appeared to run parallel to changes in MTA levels, this metabolite did not decrease significantly with Age, or across all comparisons, and showed a high fold-increase in males (compare N5 to N4). However, high outliers could also have affected these results.

### Carbohydrate and Markers of Energy Metabolism

**Figure 6:**
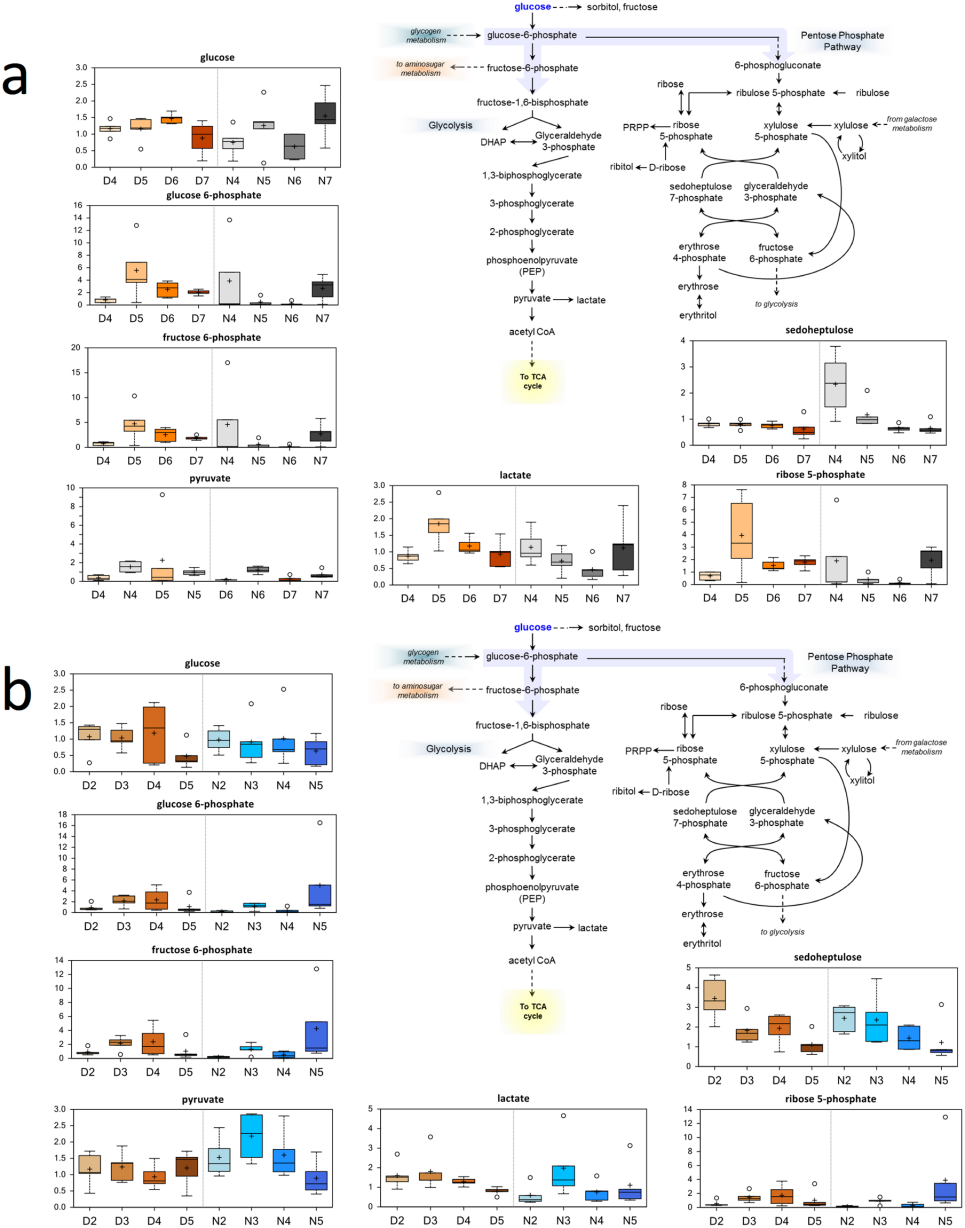
Differences in the abundances of metabolites involved in glucose metabolism in flight muscle of *Manduca sexta* across Diel Time and Age. (**a**) Female, (**b**) Male. The *x*-axis represents age post-eclosion at Day (D) and Night (N). The *y*-axis box plots indicate the scaled intensity mean (+) and median (−) values: upper and lower ranges of boxes indicate upper and lower quartiles, respectively; upper and lower whiskers indicate the maximum and minimum distributions of the data; small circles represent outlying data points. The adjusted *P* values for the significant comparisons are presented in Tables S1 and S2.

Glucose supports a variety of physiological processes, including energy generation, fatty acid synthesis, protein glycosylation, and nucleotide biogenesis. Increases in glucose (females, compare N7 to N6) and high fold-increases in fructose and mannitol or sorbitol (females, compare N7 to N6) reflect increased glucose availability at these time points (Fig. 6a). In females (compare N7 to N6), increased glucose availability may reflect increased glycogen breakdown or use (maltotetraose, maltotriose, and maltose were elevated) to meet glycolytic demand. Upstream glycolytic metabolites (glucose 6-phosphate, fructose-6-phosphate) increased in several comparisons (Fig. 6a,b) in both females (compare N7 to N6) and males (compare N5 to N4). An increase in the 3-carbon intermediate 3-phosphoglycerate in females (compare D5 to D4) and males (compare D5 to D4) suggests increased glycolysis. Finally, increases in pyruvate in males (compare N3 to N2) with a non-significant increase in females (compare D5 to D4) and increases in lactate in males (compare D3 to D2) and females (compare D5 to D4 and N7 to N6) are both consistent with increased glycolysis. Observations at the final time points at Night in females (compare N7 to N6) and males (compare N5 to N4) could reflect age-related shifts in energy use.

### TCA Cycle Changes

Carbon can flow into the TCA cycle from a number of sources, including from carbohydrates and lipids (*via* conversion of acetyl-CoA to citrate), glutamine (entering as alpha-ketoglutarate), or branched-chain amino acids (entering as citrate and succinyl-CoA). Consistent with increased glycolysis with Age, citrate and isocitrate showed a trend towards increased abundance with Age in females (Fig. S3, compare N7 to N6). Males (compare N5 to N4) showed a greater magnitude increase in TCA cycle metabolites as a class, which might reflect high energy demand or TCA cycle dysfunction.

### Alterations in Lipid Metabolism

**Figure 7:**
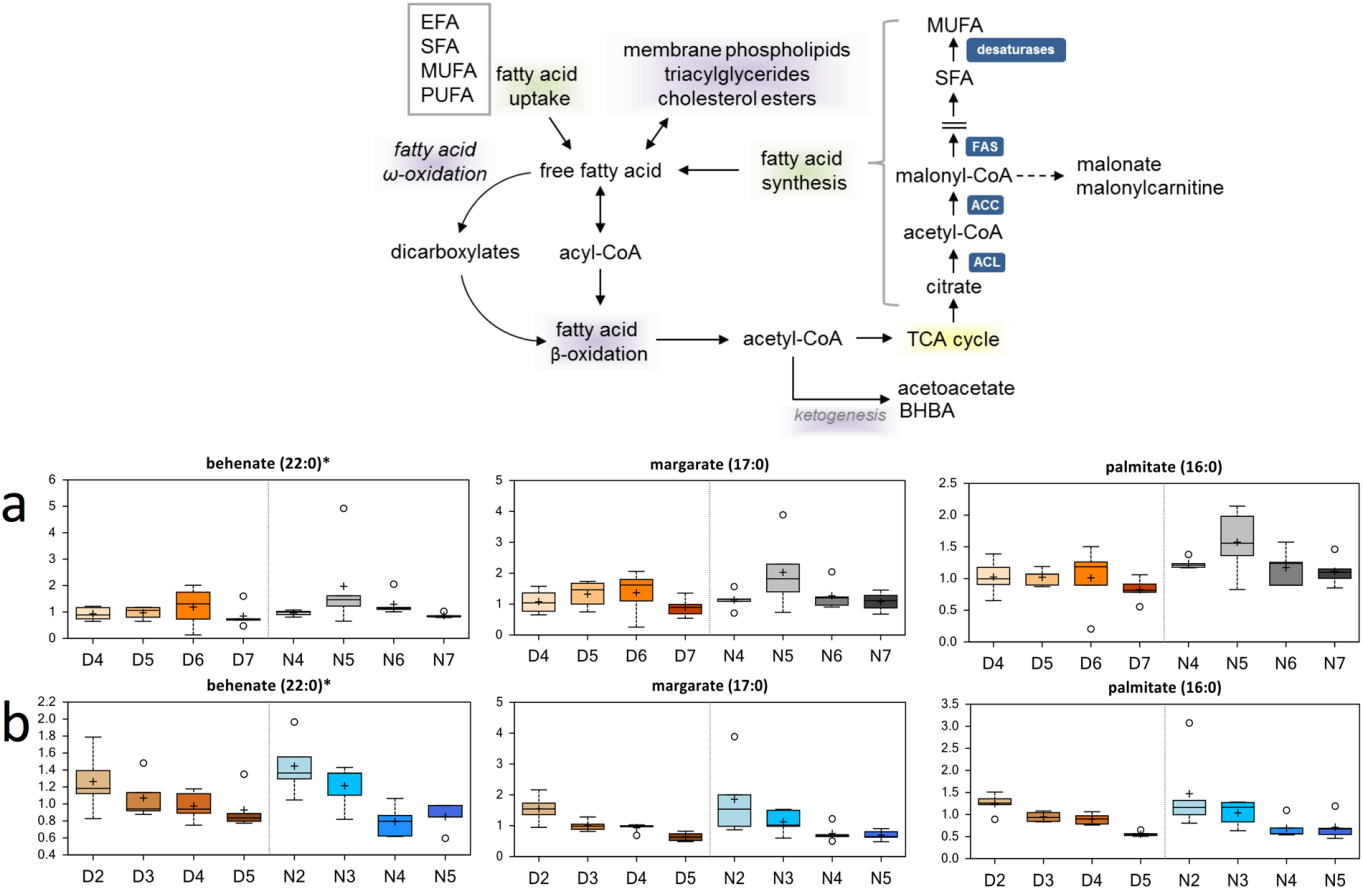
Differences in the abundances of metabolites involved in lipid metabolism in flight muscle of *Manduca sexta* across Diel Time and Age. (**a**) Female, (**b**) Male. The *x*-axis represents age post-eclosion at Day (D) and Night (N). The *y*-axis box plots indicate the scaled intensity mean (+) and median (−) values: upper and lower ranges of boxes indicate upper and lower quartiles, respectively; upper and lower whiskers indicate the maximum and minimum distributions of the data; small circles represent outlying data points. The adjusted *P* values for the significant comparisons are presented in Tables S1 and S2. *Indicates compounds that have not been officially confirmed based on a standard but we are confident in its identity.

Fatty acids (FAs) are a critical source of energy for mitochondrial oxidation and cellular ATP generation, in addition to being precursors for phospholipids and storage lipids. Long-chain fatty acids (10-heptadecenoate, 10-nonadecenoate, arachidate, eicosenoate, palmitate, and palmitoleate were significantly elevated during nighttime in females (Table S1). The abundances of several lysolipids (1-oleonyl-GPE (18:1), 1-palmitoyl-GPE (16:0), and 1-stearoyl-GPE (18:0)) significantly decreased in females with Age, which could reflect increased use to support fatty acid demand (Fig. S4). Decreases in the abundances of diacylglycerols (palmitoyl-linoleoyl-glycerol (16:0/18:2) [2], palmitoyl-olenoyl-glycerol (16:0/18:1) [1], palmitoyl-olenoyl-glycerol (16:0/18:1) [2], and stearoyl-linoleoyl-glycerol (18:0/18:2) [2]) over Diel Time or due to Age in females could also indicate changes in triacylglycerol use, while glycerol levels significantly decreased with Age in males (Table S2). Levels of behenate, margarate, oleate/vaccenate, palmitate, and stearate in males showed clear significant decreases associated with aging (Fig. 7). Interestingly, the abundances of a number lysolipids and sphingolipids significantly decreased in both Sexes with Age (Tables S1, S2, Figs S4, S5).

Long-chain fatty acids must be conjugated to carnitine for efficient transport across the mitochondrial membrane. Therefore, increases in the abundances of acylcarnitines (3-hydroxybutyrylcarnitine, behenoylcarnitine; Table S1) in females suggest age-associated changes in *beta*-oxidation. Males also showed significant decreases in the abundance of acylcarnitines, such as 3-hydroxybutyrylcarnitine, acetylcarnitine, and lignoceroylcarnitine with age (Table S2). The abundance of the ketone body 3-hydroxybutyrate (BHBA) did not trend in the same direction – levels of this metabolite significantly changed between Day and Night in females, and significantly decreased in older Age males, which could reflect differences in oxidative use between these conditions and stages.

### Biotin Metabolism Increases in Advanced Age Muscles

**Figure 8:**
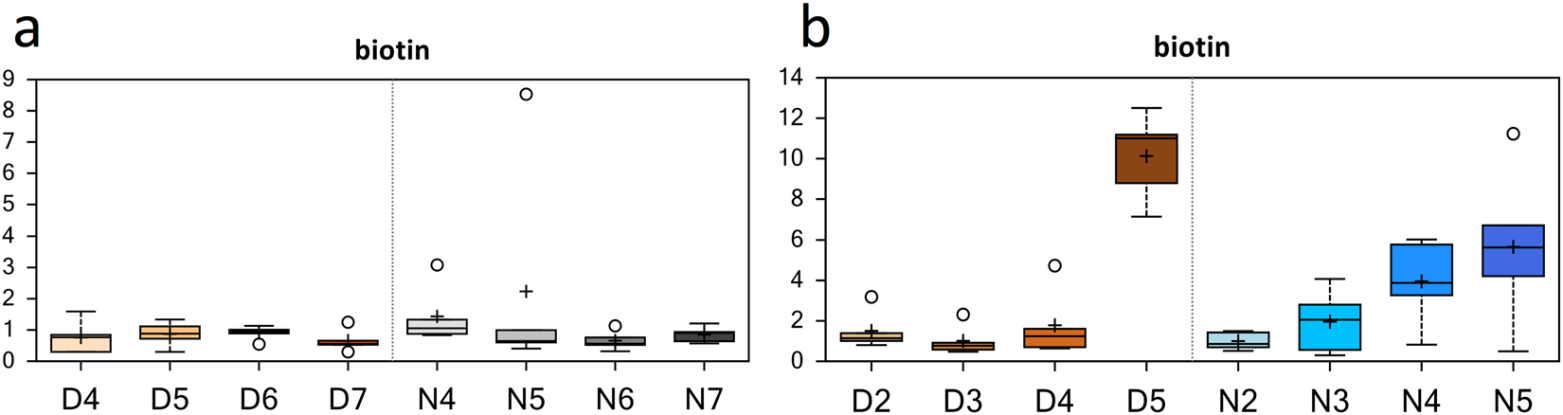
Differences in biotin abundances in flight muscle of *Manduca sexta* across Diel Time and Age. (**a**) Female, (**b**) Male. The *x*-axis represents age post-eclosion at Day (D) and Night (N). The *y*-axis box plots indicate the scaled intensity mean (+) and median (−) values: upper and lower ranges of boxes indicate upper and lower quartiles, respectively; upper and lower whiskers indicate the maximum and minimum distributions of the data; small circles represent outlying data points. The adjusted *P* values for the significant comparisons are presented in Tables S1 and S2.

Of the 18 significantly (*p* < 0.05) enriched metabolic pathways that showed greater than twofold changes in metabolite abundances, biotin metabolism was enriched 8.23-fold (Fig. S1b). Interestingly, although biotin metabolism was also one of the 75 significantly changed pathways in advanced Age males (*p* < 0.001, Fig. 8b, Table S2), this change was not detected in females (Fig. 8a, Table S1).

### Dipeptide Metabolism

The abundances of dipeptides (glycylleucine, histidylalanine, isoleucylglycine, leucylglycine, lysylleucine, phenylalanylalanine, phenylalanylglycine, tryptophylglycine, valylglycine, and valylleucine) significantly increased as a class in females, which could suggest increased protein catabolism in muscle, which may supplement energy demand or possibly reflect degenerative changes in muscle (Table S1). The abundances of branched-chain amino acid catabolites such as BHBA and butyrylcarnitine, both of which increased in nighttime samples, showed some consistent age-related changes that might support shifts in energetics. In particular, the abundances of acetylated forms of the BCAAs (N-acetylisoleucine, N-acetylleucine, and N-acetylvaline) were significantly higher during Night periods in females (Table S1).

### Redox Homeostasis

Decreases in the abundances of several *gamma*-glutamyl amino acids (*gamma*-glutamyltryptophan) were observed in females (compare D7 to D6), which could reflect changes in glutathione availability. The abundance of cysteine-glutathione disulfide also decreased), and that of ophthalmate, a biomarker of glutathione synthase activity, differed significantly different between Day and Night periods (Fig. S6). One unexpected result was that the abundance of only reduced glutathione (GSH), but not oxidized glutathione (GSSH), was significantly elevated during daytime in males. Females show much more dramatic shifts in glutathione abundances (both GSH and GSSH) over Diel Time and Age, but these changes were not significant.

## Discussion

In the present study, an untargeted metabolomics time course of flight muscle in the hawk moth, *Manduca sexta*, was performed to characterize the concerted biochemical and metabolic changes that occur during advancing age, as well as to evaluate the utility of the *M*. *sexta* system for molecular dissection of muscle aging. We provide the first detailed compendium of the metabolic alterations that occur in senescing muscle across the lifespan of *M. sexta*. This compendium will provide mechanistic insights into how these combined alterations progress over time and lead to age-related declines in muscle performance and function.

One interesting metabolic alteration highlighted by our results was biotin levels were significantly elevated in the flight muscle of advanced age male moths (Figs S1b, 8), but similar increases were not observed in advanced age females. One likely explanation for this is that the females sampled at D7 are not as old physiologically as males sampled on D5, possibly because females feed and thus live longer than males sampled at D5. We hypothesize that the observed difference in biotin levels between males and females could be due to advanced age-related deceleration of energy metabolism^38^. Hence, biotin would accumulate due to muscle cell death, other signs of this were also apparent in the metabolomics profiles of males (Fig. 3d). Biotin is a cofactor for acetyl-CoA carboxylase (isoforms α and β; E.C. 6.4.1.2), pyruvate carboxylase (E.C. 6.4.1.1), propionyl-CoA carboxylase (E.C. 6.4.1.3), and 3-methylcrotonyl-CoA carboxylase (E.C. 6.4.1.4), which are essential in the metabolism of glucose, amino acids, and fatty acids^39^. In addition, biotin regulates gene expression through histone modifications^39^. Biotin accumulates in adipose tissues during human aging^40^, and this accumulation is associated with the expression and activity of sirtuin 1 (*SIRT1*). Decreased expression and activity of the *SIRT1* gene in adipose tissue subsequently affects energy metabolism in other tissues, such as muscle, *via* both biotin-dependent carboxylases (non-genetic) and biotinylation of histones (epigenetic) mechanisms^40^. Given that both humans and fruit flies express these same four biotin-dependent carboxylases^41^, biotin accumulation in muscle could serve as a novel biomarker for muscle aging. To our knowledge, we are the first to track and report elevated biotin accumulation in advanced age muscles of any organism. Biotin is widely recognized for its metabolic and transcriptional roles. However, the molecular details surrounding the exact cellular function(s) of biotin, especially its role in muscle aging, are not clear^38^ and thus warrant further studies.

Another metabolic alteration highlighted by our results was a shift in the levels of collagen-associated metabolites with Age. We observed decreases in collagen-associated metabolites in both female and male moths of advanced age (Fig. 4). Decreased collagen turnover can affect muscle function during aging^42^, and can result in reduced muscle mass over time^43^. Decreased collagen turnover has also been observed in aging skeletal muscle in humans^42^. Indeed, age-related changes in the extracellular matrix in muscle can contribute to increased stiffness and reduction of the amount force that can be generated by contracting muscle fibers^42^. Collagen-associated metabolites, such as 5-hydroxylysine, may therefore serve as a useful biomarker for evaluating changes in muscle biology associated with aging (*see below*).

A second metabolic pathway highlighted by our results was the fatty acid metabolism pathway (Figs S1b, 7). We observed decreased fatty acid metabolism associated with Age in both Sexes. Lipid oxidation typically provides support for energy demands during moderate exercise^44^. Shifts away from *beta*-oxidation suggest increased energy demands in aging muscle, subtle changes in mitochondrial function, or shifts toward glycolysis during advanced age^32,34^. As previously mentioned, decreased fatty acid oxidation with shifts toward increased glycolysis have also been observed in aging rodent muscle^32,33^. Interestingly, females show increased fatty acid metabolism at Night, but similar shifts were not observed during the Day. This lack of daytime increases in fatty acid metabolism reflects low energy demand in these tissues during their inactive period, and suggests a possible circadian pattern of regulation in this pathway^45^. We argue that males do not show this diel shift in fatty acid metabolism because, unlike females, they tend not to feed during their active period^43^ and because feeding moths use fatty acids from nectar as a metabolic fuel^46^. The fact that the abundance of long-chain fatty acids does not change in male moths between Day and Night adds further evidence that male hawk moths do not generally feed. Thus, unlike females, males do not acquire new fatty acids by feeding, as can be seen in the lack of diel shifts in their metabolic profiles (Fig. 3c, Table S2), but rather use only resources already present in their larval state. In fact, the significant decreases in the abundance of a large number of lysolipids (Fig. S4, Table S2) in males with Age likely reflects increased use of these lipids to support energetic demands that result from the fact that they do not feed. Notably, although males showed increased glutathione metabolism (i.e., GSH) during daytime compared to nighttime (Fig. S6), such shifts were more noticeable in females, but were not statistically significant. In addition, the abundances of 5-oxoproline, ophthalmate, and putrescine increased significantly in females during the nighttime compared to the daytime (Figs 5, S6). Glutathione activity is elevated in feeding moths *via* activation of the pentose phosphate pathway (PPP) to cope with oxidative damage in muscle that results from flight^47^. Our results suggest that PPP activity might decrease during advanced age, as indicated by the significant decreases in the abundance of sedohepulose^48^ in both Sexes (Fig. 6). Sphingolipids and ceramides represent other fatty acid species that decrease with Age in both Sexes (Fig. S5). Decreases in the abundance of these metabolites in muscle cells might protect against muscle cell dysfunction and death^49,50^. Indeed, reduced anabolic signaling in skeletal muscle of aged mice has been linked to accumulation of intramuscular ceramides and diacylglycerols^51^.

Another pathway affected in older moths is polyamine metabolism (Figs S1b, 5). We found age-related decreases in the abundances of SAMe and MTA in flight muscle of both female and male moths of advanced age, which indicates aging-related changes in polyamine metabolism. No consistent changes in the levels of polyamines (spermidine) that might be associated with aging were apparent, although pools of these metabolites might be in steady state. Therefore, changes in MTA abundance might reflect differences in polyamine metabolism related to antioxidant function in muscle maintenance and regeneration. Because polyamines are negatively charged polycations, they can interact with DNA, RNA, or proteins, and can thus be involved in aging, stress, growth, and disease responses in both plants and animals^52^. In animals, polyamine levels are known to decrease with age^53,54^. Spermidine and spermine levels decreased in the muscles of 3- to 26-week-old mice^53^. Polyamine levels also decreased in thymus, spleen, ovary, liver, stomach, lung, kidney, heart, and liver in 3- to 26-week-old mice, but not in pancreas, brain, or uterus. Nishimura *et al*.^53^ suggested that the maintenance of polyamine levels might be important for the function of the pancreas, brain, and uterus in 3- to 26-week-old mice. One important function of polyamines is regulation of energy and glucose metabolism^54,55^. As such, age-related decreases in muscle polyamine levels might result in gradual declines in both muscle maintenance and metabolism and lead to deterioration of muscle function and performance with advanced age.

One of the goals of our study was to identify and track novel muscle aging biomarkers, and seek insights into the regulatory aspects of muscle aging. Our time-series approach identified notable changes in the metabolism of the cofactor biotin, polyamines (SAMe and MTA), and modified amino acids (5-hydroxylysine). We also tracked metabolite sets that changed across Diel Time and during aging, including fatty acid and glucose metabolism. As discussed above, changes in the levels of modified amino acids, polyamines, and fatty acid derivatives are of high predictive value for identification and separation of middle aged from advanced age moths.

Adult *M. sexta* females in the present study had longer life expectancy than did males, whether they were fed or unfed, consistent with Ziegler’s^43^ observations and also reflects their natural life history. Wild *M. sexta* are distributed across the Americas and are active and reproduce mainly during the summer monsoon season in the Sonoran Desert of the Southwestern United States^56,57^. Hawk moths are in diapause for most of the year, but can produce a spring cohort if environmental conditions are favorable. Ziegler^43^ reported that laboratory-reared adults feed very irregularly and that females are more likely to feed whether they were freely offered nectar or were individually hand fed. Our results also indicate that while females generally feed, males generally do not, which is reflected in the observed absence of shifts in male metabolic profiles due to Diel Time in the present study (Fig. 3a,c). Not surprisingly, feeding increased the number of eggs laid. Unfed females laid an average of 123 eggs and fed females laid an average 1,143 eggs^43^. Sasaki and Riddiford^58^ reported similar results. Thus, we suspect that wild females might have similar feeding behavior as lab-reared females and although they might still be able to reproduce when food is not available, they are less fecund.

*Manduca sexta* is already a prominent animal model for neurobiology, endocrinology, flight mechanics, larval nicotine resistance, immune function, and development, with proven experimental strengths that could be combined with those of other models of aging to expand our understanding of how muscles senesce^59^. Further, our results show that *M. sexta* is an excellent, complementary, non-vertebrate, animal model of muscle aging. The use of non-vertebrate animal models for research is facile and their use is often more ethically acceptable. Such organisms have short-life cycles, low husbandry costs, and are inexpensive models for a variety of human skeletal muscle diseases^60,61^ because they have genes homologous to those identified in human or murine models. For example, the protein encoded by a gene homologous to the *ACTN3* gene in *Drosophila*^61^ reportedly maintains muscle performance in humans^62,63^ and rodents^64^. Further, the short lifespan of *M. sexta* is conducive to molecular dissection of muscle aging at high temporal resolution, as we have shown. Lastly, the flight muscle of *M. sexta* is similar to vertebrate skeletal muscle, both functionally because it is synchronous and metabolically because it is endothermic^65–67^. This physiological nexus with vertebrates might make the hawk moth flight muscle a more accurate model for vertebrate muscle aging at the biochemical and molecular levels. Studies of this non-vertebrate model of muscle aging will provide a fundamental understanding of the senescence process that could result in translational applications^68,69^.

In summary, our global metabolomic study comparing flight muscle from male and female *M. sexta* at multiple time points, with a focus on age-related changes, identified a number of differences between sexes and time points, including changes in the abundances of metabolites related to energetics, extracellular matrix turnover, and polyamine metabolism. The abundances of metabolites derived from collagen turnover, such as 5-hydroxylysine, significantly decreased over time, consistent with observations in mammalian models. Similarly, levels of SAMe and MTA significantly decreased with Age, which reflected changes in polyamine metabolism. Changes in glycolytic metabolites suggest shifts toward increased glycolysis with Age, with differences in the abundances of lysolipids and acylcarnitines reflecting decreasing activity of *beta*-oxidative pathways. The progression of these combined alterations over time is related to and might influence or modulate age-related declines in muscle performance and function in *M. sexta*. Interestingly, metabolic changes in daytime samples were subtler, which could reflect decreased energy requirements during the inactive period for nocturnal insects and could be under circadian control. Given that age-related differences in the changes in energetic pathways are subtler during the Day, it might be interesting to see whether disruption of circadian cycles in *M. sexta* would result in any differential effects on metabolites related to muscle aging. Although the lifespan of *M. sexta* is relatively short, our metabolic profiles across Diel Time and Age suggest that these moths can meet the high metabolic demands of flight, and at the same time minimize muscle cell damage due to high rates of metabolic activity as they age. Finally, our approach using extensive global ‘omics analyses of flight muscle in *M. sexta* across both Diel Time and Age will provide a fundamental basis for future molecular dissection of the regulation of muscle aging in this model species and will also allow extensive cross-species comparisons^70^.

## Methods

### Animals

Fertilized eggs of *Manduca sexta* (Sphingidae, Lepidoptera) were obtained from a laboratory colony at the University of Arizona^71^. Larvae that subsequently emerged were reared with *ad lib* artificial diet for moths^72^. Eggs, larvae, and adults were reared under 16 h light/8 h dark at 25 °C and 60% humidity. Four adult moths were housed together per 598 cm^3^ cage (28 cm × 28 cm × 56 cm (L × W × H)) comprised of two stacked Bug Dorms (BioQuip Products, Rancho Dominguez, CA, USA). Adult moths in all treatments were fed with *ad lib* artificial nectar (Educational Science, League City, Texas, USA).

### Lifespan determination

Dates of eclosion and death were recorded for each individual. Eclosed moths within each sex were randomly assigned to one of four types of treatment cage: those containing only female moths fed *ad lib*, only female moths unfed, only male moths fed *ad lib*, or only males only unfed. Each adult was individually marked with a small drop of nail polish to identify its treatment group. Emerged moths were weighed <12 h post-eclosion and were checked twice daily until death, at which time they were weighed again. Moths were collected once per day in the morning and lifespans were calculated in full days.

### Time-series sampling for metabolomics analyses

Based on results from the lifespan study, middle Age in male moths was identified as Day 2 (D2) and advanced Age as day 5 (D5) post-eclosion. In female moths, middle Age was identified as Day 4 (D4) and advanced Age as Day 7 (D7) post-eclosion. The dorsolateral flight muscles of adults were collected at time points (see below) beginning at middle Age until advanced Age during photophase (moth’s inactive period) and scotophase (moth’s active period). Muscle collection for photophase started at 0900 h and for scotophase started at 2100 h (after ~1 h of activity). Adult moths were euthanized by decapitation and their flight muscles quickly dissected and flash frozen in liquid nitrogen (<90 sec from time of death to flash freezing) then stored at −80 °C until metabolite extraction. Animals used for muscle metabolic profiling were unmated animals housed four per cage with *ad lib* artificial nectar. Five biological replicates from each sex were collected at each time point.

### Global metabolic profiling

For metabolite extractions, flight muscle was first ground to a fine powder under liquid nitrogen and 20 mg of powdered muscle was used for the unbiased global metabolic profiling performed by Metabolon (Durham, NC, USA). Briefly, each 20-mg muscle tissue sample was extracted at a 1:5 ratio in methanol plus recovery standards using an automated MicroLab STAR system (Hamilton Company, Salt Lake City, UT, USA). The extracted samples were split into equal parts for analysis on two ultra-high performance liquid chromatography/tandem mass spectrometry (UHLC/MS/MS^2^) instruments optimized either for basic species or for acidic species^73^.

### Statistics and Bioinformatics

We estimated lifespan with a generalized least-squares fit by REML (Restricted Maximum Likelihood) model using the nlme package in *R* (version 3.1-128^74^), with Age as the dependent variable and Sex and feeding treatment (Fed or Unfed) as independent variables. *Post hoc* multiple comparisons were performed as pairwise comparisons with Tukey corrections using the *R* packages contrast, version 0.19^75^ and multcomp^76^.

Metabolomics data in figures are presented as mean ±SEM or as box- and-whisker plots indicating the sample minimum, lower quartile, median, upper quartile, and maximum with outliers represented as small circles. When a metabolite was below the threshold of detection, data was imputed as the minimum detected quantity for that metabolite in this project. Resulting data were log (base2)-transformed to normalize and then initially analyzed using a three-way ANOVA (including Sex, Time-of-Day, and Age as main effects with interactions). Unsupervised HCA was performed using complete linkage and Euclidian distance to assess sample similarity. RFA, which was performed to identify biomarkers for muscle aging, is a supervised classification technique based on an ensemble of decision trees^77^. This approach requires no parametric assumptions or variable selection, does not overfit the data, is invariant to transformation, and is fairly easy to implement in *R* (*see* Mitchell^78^ for analysis flow).

The web-based metabolomics data processing tool, MetaboAnalyst 3.0, was used to provide overviews of metabolite data (i.e., Orthogonal Partial Least Squares Discriminant Analysis (OPLS-DA)) and two-way analysis of variance (ANOVA). See http://www.metaboanalyst.ca/faces/home.xhtml for detailed methodology. Briefly, for both OPLS-DA and ANOVA, metabolite data were Pareto scaled and OPLS-DA was performed using the orthoPLS-DA module. ANOVA was performed using the two-way (between subjects) ANOVA module. An adjusted *P* value of 0.05 or less was considered significant, with the false discovery rate used to correct for multiple comparisons. All reported *P*-values from ANOVA are adjusted *P*-vales. Heatmap visualization of significant metabolite abundances was developed from hierarchical clustering using complete linkage and Euclidian distance. Colors in the heatmaps reflect relative metabolite abundances: red represents metabolite abundances higher than the mean and blue represents metabolite abundances lower than the mean. OPLS-DA models were permuted 1,000 times to assess model robustness.

### Data availability statement

All data generated/analyzed in this study are included in this published article (and its Supplementary Information files), or will be provided upon request.

## Electronic supplementary material

Supplementary Information
