# Supplementary material for: A procession of metabolic alterations accompanying muscle senescence in Manduca sexta
Source: Sci Rep. 2018 Jan 17;8:1006. doi: 10.1038/s41598-018-19630-5 (PMC5772441; doi:10.1038/s41598-018-19630-5)
Supplement: Supplementary file 1 — Supplementary Information [file 41598_2018_19630_MOESM1_ESM.pdf]

SUPPLEMENTARY MATERIAL

A procession of metabolic alterations accompanying muscle senescence in *Manduca sexta*

Bernard W. M. Wone*, Jason M. Kinchen, Elana R. Kaup, Beate Wone

*Correspondence and requests for materials should be addressed to B.W.M.W. (email: b.wone10@gmail.com)

a

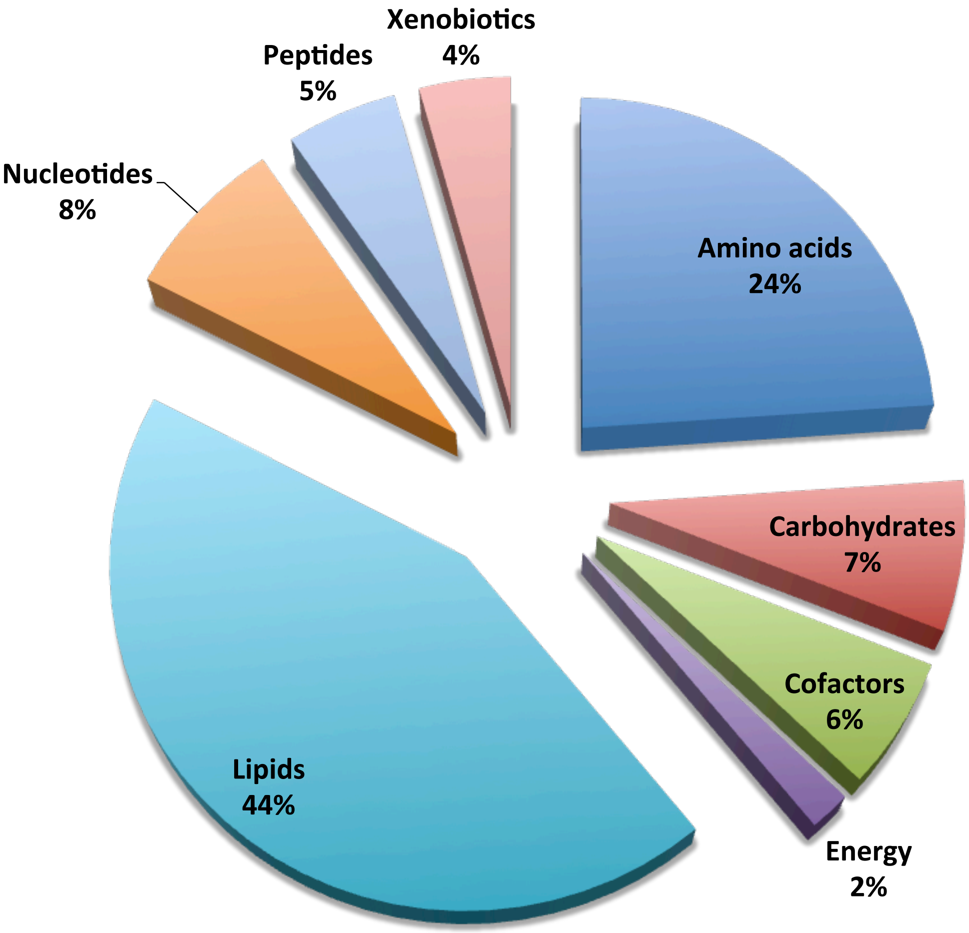
Xenobiotics
4%
Peptides
5%
Nucleotides
8%
Amino acids
24%
Carbohydrates
7%
Cofactors
6%
Energy
2%
Lipids
44%

b

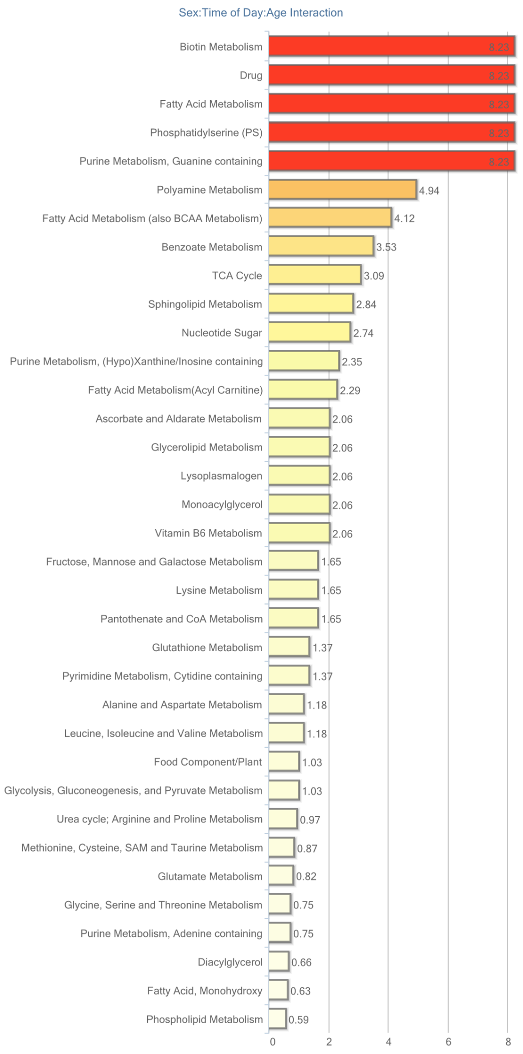
Sex:Time of Day:Age Interaction
Biotin Metabolism 8.23
Drug 8.23
Fatty Acid Metabolism 8.23
Phosphatidylserine (PS) 8.23
Purine Metabolism, Guanine containing 8.23
Polyamine Metabolism 4.94
Fatty Acid Metabolism (also BCAA Metabolism) 4.12
Benzoate Metabolism 3.53
TCA Cycle 3.09
Sphingolipid Metabolism 2.84
Nucleotide Sugar 2.74
Purine Metabolism, (Hypo)Xanthine/Inosine containing 2.35
Fatty Acid Metabolism(Acyl Carnitine) 2.29
Ascorbate and Aldarate Metabolism 2.06
Glycerolipid Metabolism 2.06
Lysoplasmalogen 2.06
Monoacylglycerol 2.06
Vitamin B6 Metabolism 2.06
Fructose, Mannose and Galactose Metabolism 1.65
Lysine Metabolism 1.65
Pantothenate and CoA Metabolism 1.65
Glutathione Metabolism 1.37
Pyrimidine Metabolism, Cytidine containing 1.37
Alanine and Aspartate Metabolism 1.18
Leucine, Isoleucine and Valine Metabolism 1.18
Food Component/Plant 1.03
Glycolysis, Gluconeogenesis, and Pyruvate Metabolism 1.03
Urea cycle; Arginine and Proline Metabolism 0.97
Methionine, Cysteine, SAM and Taurine Metabolism 0.87
Glutamate Metabolism 0.82
Glycine, Serine and Threonine Metabolism 0.75
Purine Metabolism, Adenine containing 0.75
Diacylglycerol 0.66
Fatty Acid, Monohydroxy 0.63
Phospholipid Metabolism 0.59
0
2
4
6
8

Figure S1. Global metabolome of flight muscle in senescing *Manduca sexta*. (**a**) Functional categorization of 535 identified metabolites detected across Diel Time from middle to advanced Age. (**b**) Metabolic pathways that were significantly enriched ($P < 0.05$).

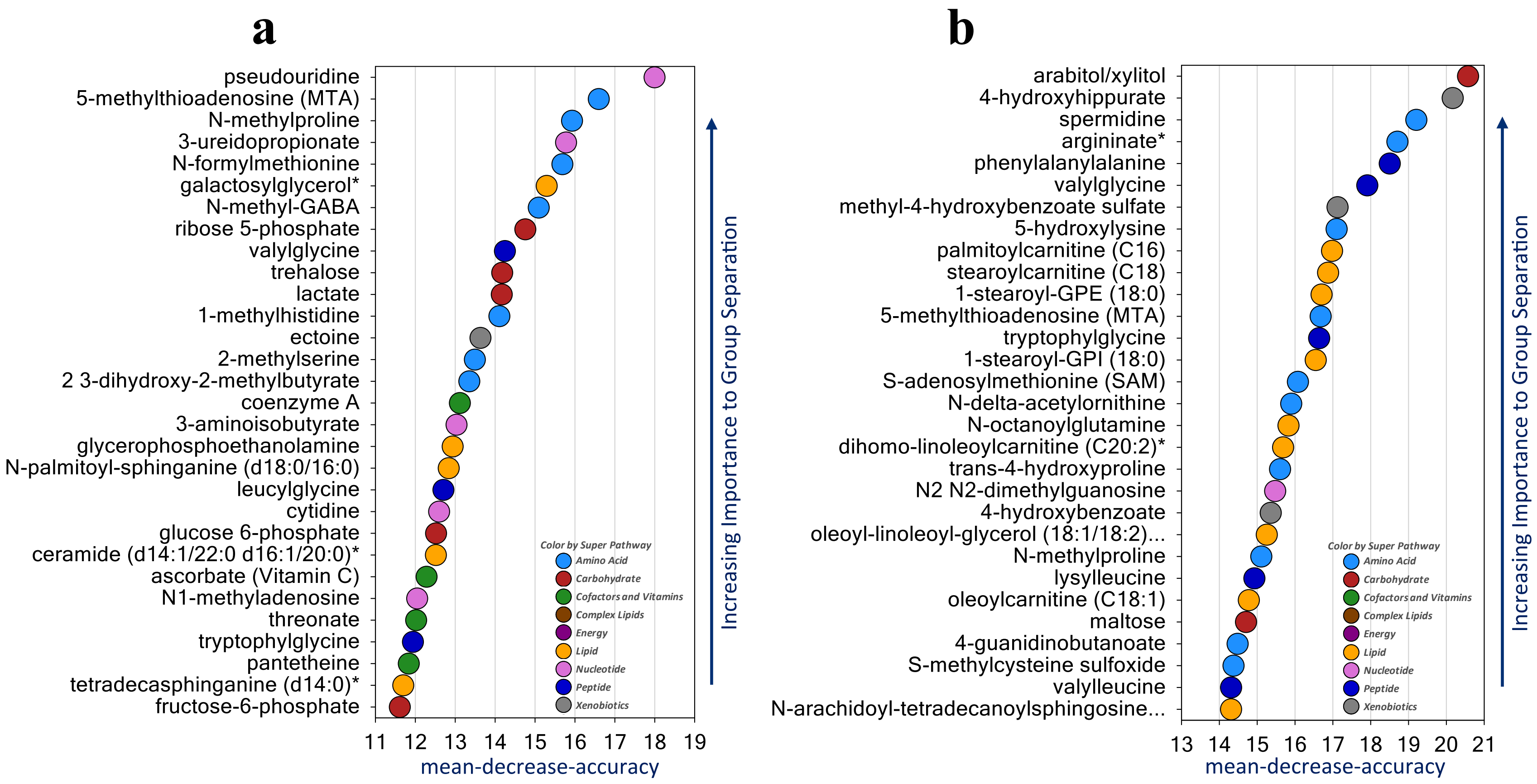
a
pseudouridine
5-methylthioadenosine (MTA)
N-methylproline
3-ureidopropionate
N-formylmethionine
galactosylglycerol*
N-methyl-GABA
ribose 5-phosphate
valylglycine
trehalose
lactate
1-methylhistidine
ectoine
2-methylserine
2 3-dihydroxy-2-methylbutyrate
coenzyme A
3-aminoisobutyrate
glycerophosphoethanolamine
N-palmitoyl-sphinganine (d18:0/16:0)
leucylglycine
cytidine
glucose 6-phosphate
ceramide (d14:1/22:0 d16:1/20:0)*
ascorbate (Vitamin C)
N1-methyladenosine
threonate
tryptophylglycine
pantetheine
tetradecasphinganine (d14:0)*
fructose-6-phosphate
11 12 13 14 15 16 17 18 19
mean-decrease-accuracy
Increasing Importance to Group Separation
Color by Super Pathway
Amino Acid
Carbohydrate
Cofactors and Vitamins
Complex Lipids
Energy
Lipid
Nucleotide
Peptide
Xenobiotics
b
arabitol/xylitol
4-hydroxyhippurate
spermidine
argininate*
phenylalanylalanine
valylglycine
methyl-4-hydroxybenzoate sulfate
5-hydroxylysine
palmitoylcarnitine (C16)
stearoylcarnitine (C18)
1-stearoyl-GPE (18:0)
5-methylthioadenosine (MTA)
tryptophylglycine
1-stearoyl-GPI (18:0)
S-adenosylmethionine (SAM)
N-delta-acetylornithine
N-octanoylglutamine
dihomo-linoleoylcarnitine (C20:2)*
trans-4-hydroxyproline
N2 N2-dimethylguanosine
4-hydroxybenzoate
oleoyl-linoleoyl-glycerol (18:1/18:2)...
N-methylproline
lysylleucine
oleoylcarnitine (C18:1)
maltose
4-guanidinobutanoate
S-methylcysteine sulfoxide
valylleucine
N-arachidoyl-tetradecanoylsphingosine...
13 14 15 16 17 18 19 20 21
mean-decrease-accuracy
Increasing Importance to Group Separation
Color by Super Pathway
Amino Acid
Carbohydrate
Cofactors and Vitamins
Complex Lipids
Energy
Lipid
Nucleotide
Peptide
Xenobiotics

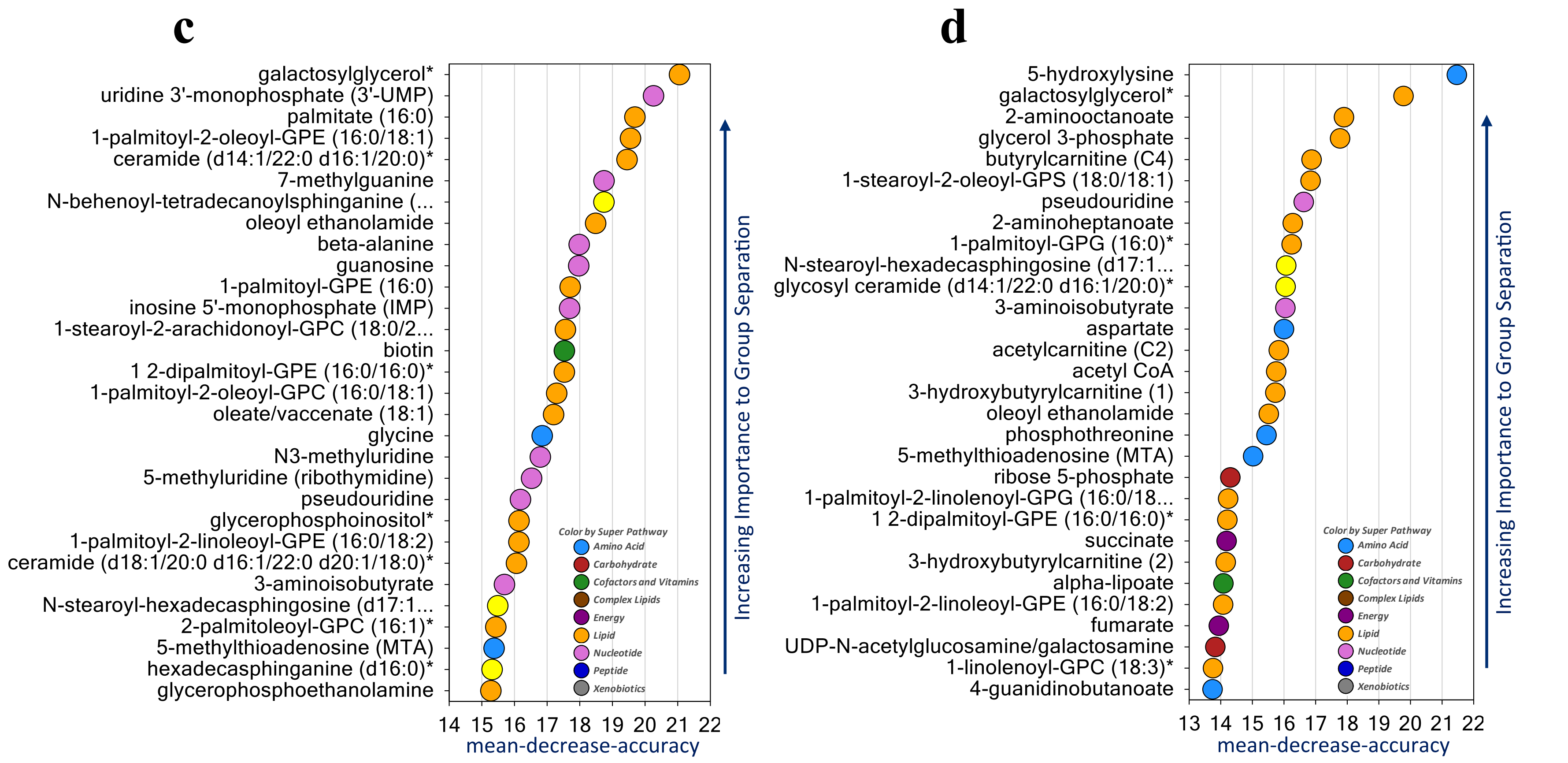

Figure S2. Identification of biomarkers for muscle aging by Random Forest Analysis of global metabolites in flight muscle of senescing *Manduca sexta*. These biochemical importance plots indicate the rankings of metabolites based on their increasing importance to group separation according to Age. We list the 30 most important metabolites to group separation according to Age and their cumulative predictive accuracy. Female: (**a**) Day gave a predictive accuracy of 50%; (**b**) Night gave a predictive accuracy of 80%. Male (**c**) Day gave a predictive accuracy of 75%; (**d**) Night gave a predictive accuracy of 60%. Colored symbols are used to indicate the super pathway to which the respective metabolite belongs. *Indicates compounds that have not been officially confirmed based on a standard but we are confident in its identity.

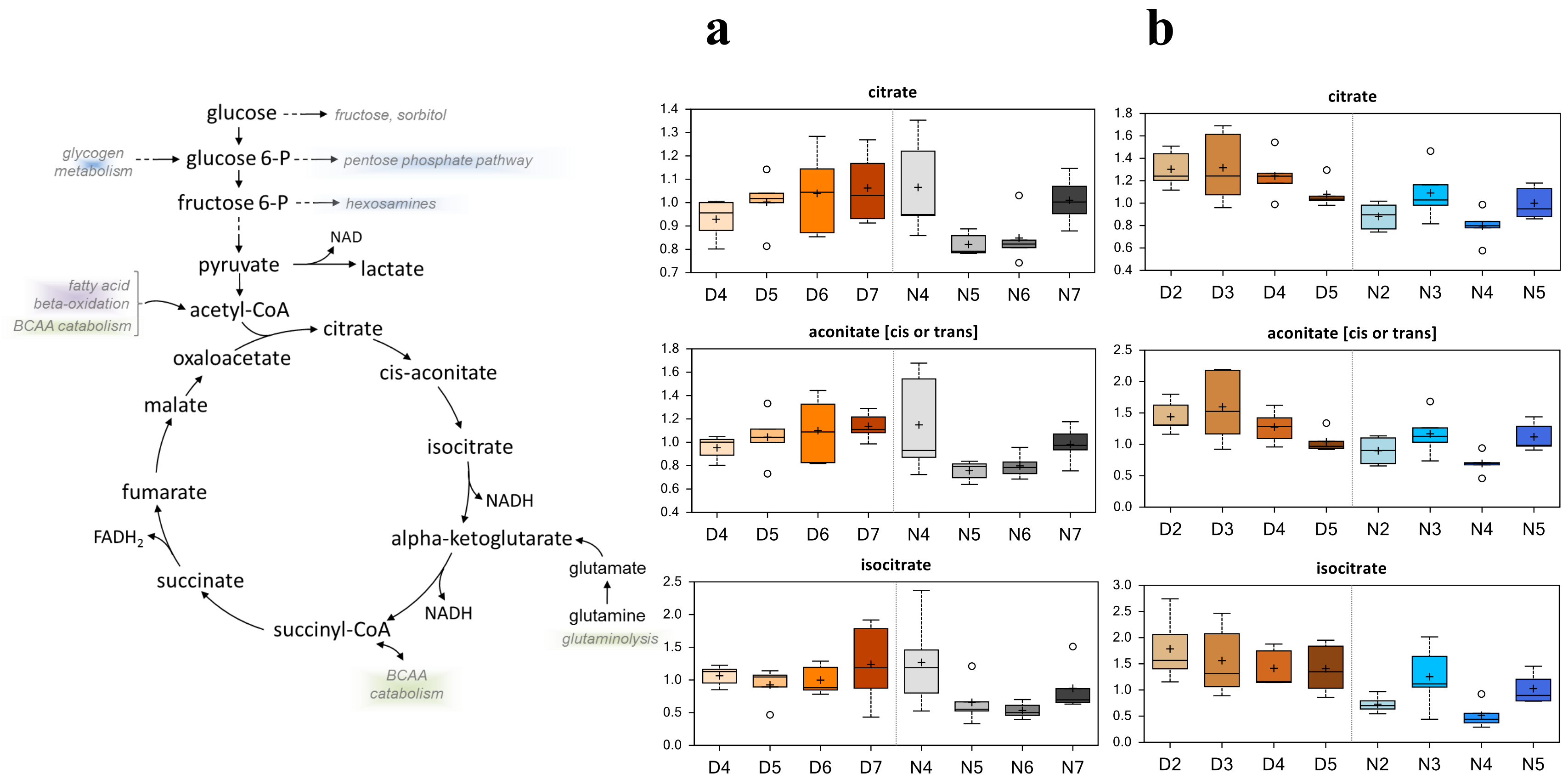

Figure S3. Differences in the abundances of metabolites involved in energy metabolism in flight muscle of *Manduca sexta* across Diel Time and Age. (**a**) Female, (**b**) Male. The *x*-axis represents age post-eclosion at Day (D) and Night (N). The *y*-axis box plots indicate the scaled intensity mean (+) and median (–) values: upper and lower ranges of boxes indicate upper and lower quartiles, respectively; upper and lower whiskers indicate the maximum and minimum distributions of the data; small circles represent outlying data points. The adjusted *P* values for the significant comparisons are presented in Tables S1 and S2.

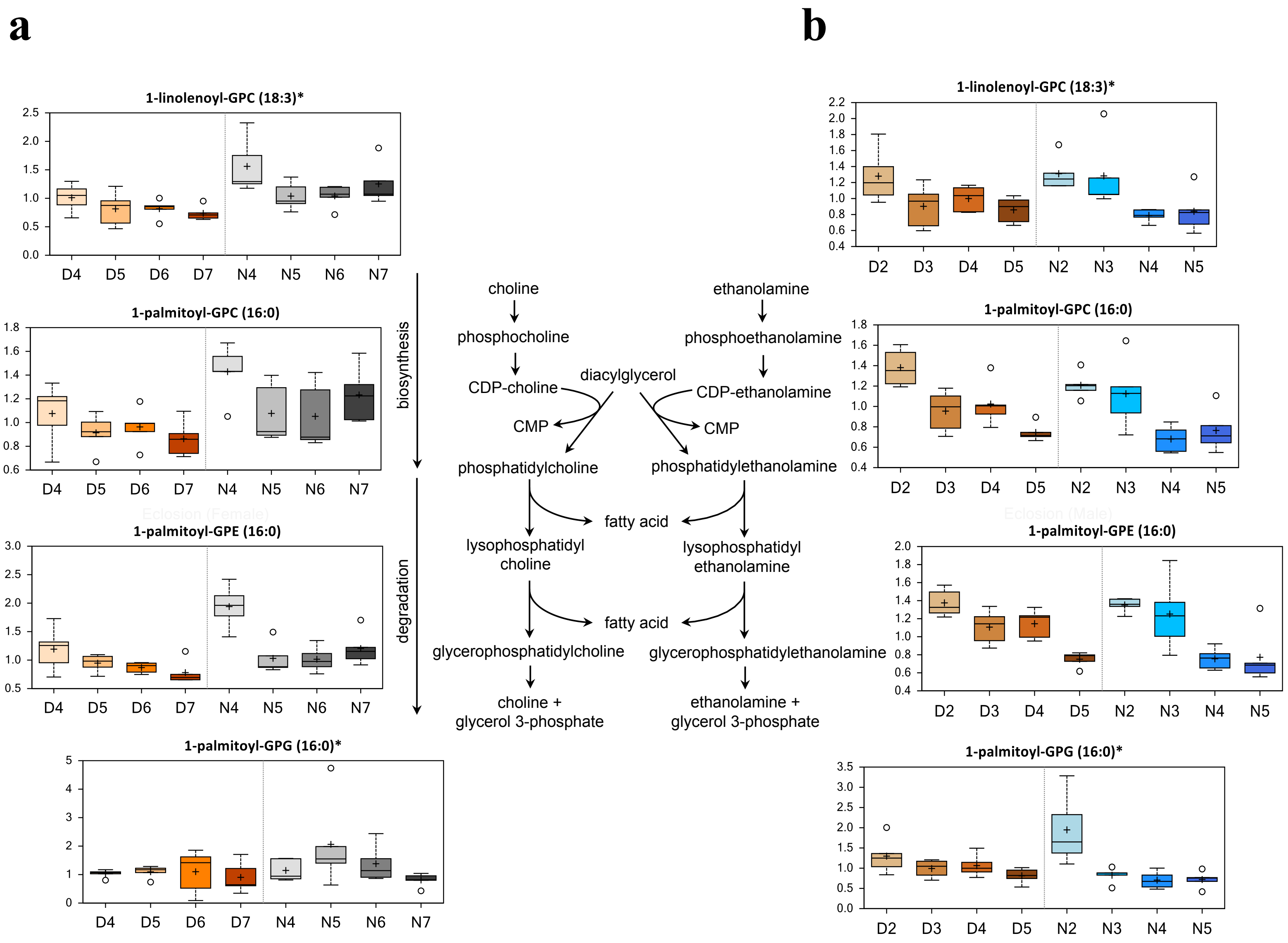
a
b
1-linolenoyl-GPC (18:3)*
1-palmitoyl-GPC (16:0)
1-palmitoyl-GPE (16:0)
1-palmitoyl-GPG (16:0)*
D4 D5 D6 D7 N4 N5 N6 N7
D2 D3 D4 D5 N2 N3 N4 N5
biosynthesis
degradation
choline
phosphocholine
CDP-choline
CMP
diacylglycerol
phosphatidylcholine
ethanolamine
phosphoethanolamine
CDP-ethanolamine
CMP
phosphatidylethanolamine
fatty acid
lysophosphatidyl choline
lysophosphatidyl ethanolamine
fatty acid
glycerophosphatidylcholine
glycerophosphatidylethanolamine
choline + glycerol 3-phosphate
ethanolamine + glycerol 3-phosphate

Figure S4. Differences in lysolipid abundances in flight muscle of *Manduca sexta* across Diel Time and Age. (**a**) Female, (**b**) Male. Not all significant lysolipids are shown; see Tables S1 and S2 for complete list. The *x*-axis represents age post-eclosion at Day (D) and Night (N). The *y*-axis box plots indicate the scaled intensity mean (+) and median (–) values: upper and lower ranges of boxes indicate upper and lower quartiles, respectively; upper and lower whiskers indicate the maximum and minimum distributions of the data; small circles represent outlying data points. The adjusted *P* values for the significant comparisons are presented in Tables S1 and S2. *Indicates compound that has not been officially confirmed based on a standard, but for which we are confident of the identity.

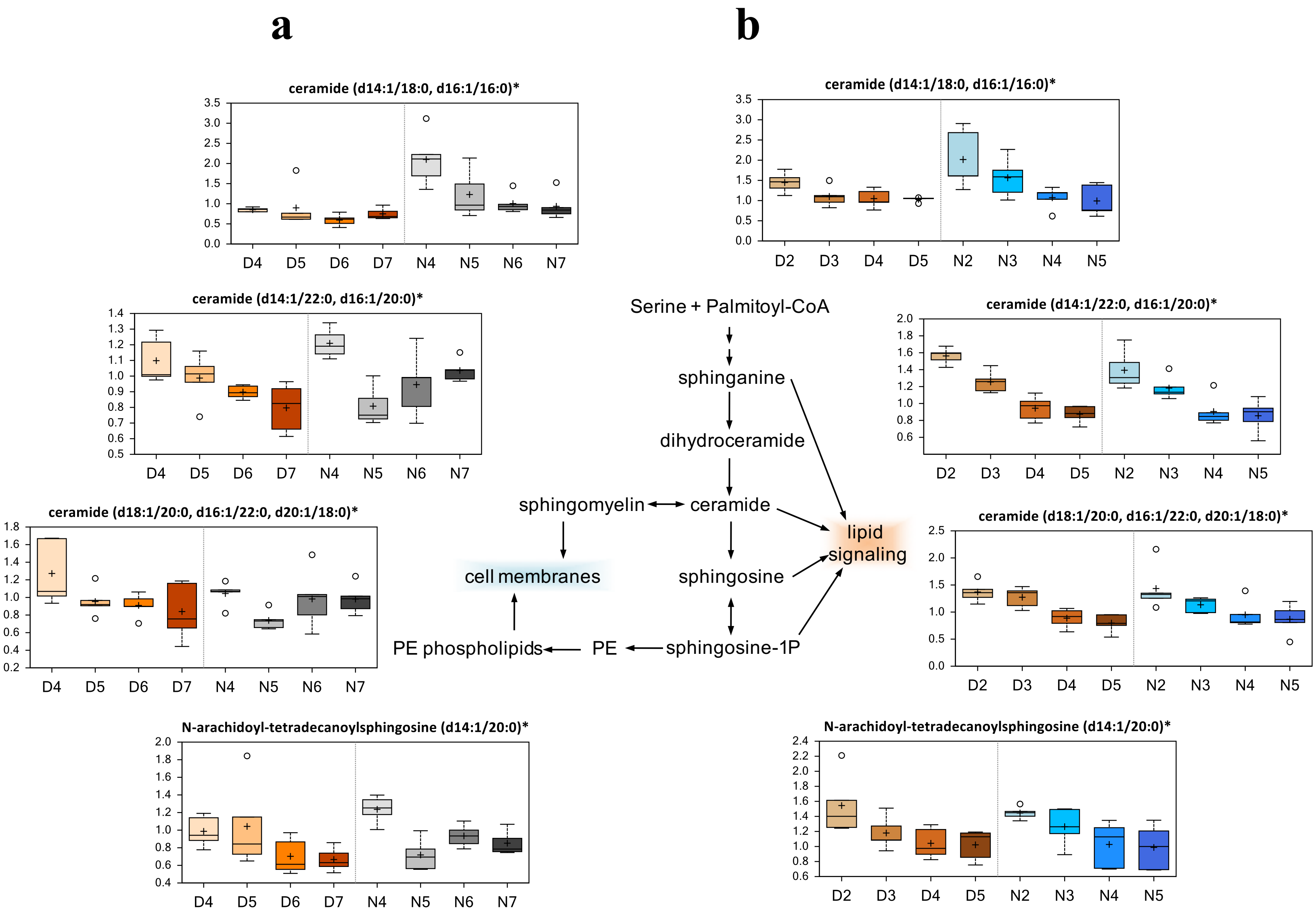
a
b
ceramide (d14:1/18:0, d16:1/16:0)*
D4 D5 D6 D7 N4 N5 N6 N7
ceramide (d14:1/22:0, d16:1/20:0)*
D4 D5 D6 D7 N4 N5 N6 N7
ceramide (d18:1/20:0, d16:1/22:0, d20:1/18:0)*
D4 D5 D6 D7 N4 N5 N6 N7
N-arachidoyl-tetradecanoylsphingosine (d14:1/20:0)*
D4 D5 D6 D7 N4 N5 N6 N7
ceramide (d14:1/18:0, d16:1/16:0)*
D2 D3 D4 D5 N2 N3 N4 N5
ceramide (d14:1/22:0, d16:1/20:0)*
D2 D3 D4 D5 N2 N3 N4 N5
ceramide (d18:1/20:0, d16:1/22:0, d20:1/18:0)*
D2 D3 D4 D5 N2 N3 N4 N5
N-arachidoyl-tetradecanoylsphingosine (d14:1/20:0)*
D2 D3 D4 D5 N2 N3 N4 N5
Serine + Palmitoyl-CoA
sphinganine
dihydroceramide
sphingomyelin
ceramide
cell membranes
sphingosine
lipid signaling
PE phospholipids
PE
sphingosine-1P

Figure S5. Differences in sphingolipid and ceramide abundances in flight muscle of *Manduca sexta* across Diel Time and Age. (**a**) Female, (**b**) Male. Not all significant sphingolipids are shown; see Tables S1 and S2 for complete list. The *x*-axis represents age post-eclosion at Day (D) and Night (N). The *y*-axis box plots indicate the scaled intensity mean (+) and median (–) values: upper and lower ranges of boxes indicate upper and lower quartiles, respectively; upper and lower whiskers indicate the maximum and minimum distributions of the data; small circles represent outlying data points. The adjusted *P* values for the significant comparisons are presented in Tables S1 and S2. *Indicates compound that has not been officially confirmed based on a standard, but for which we are confident of the identity.

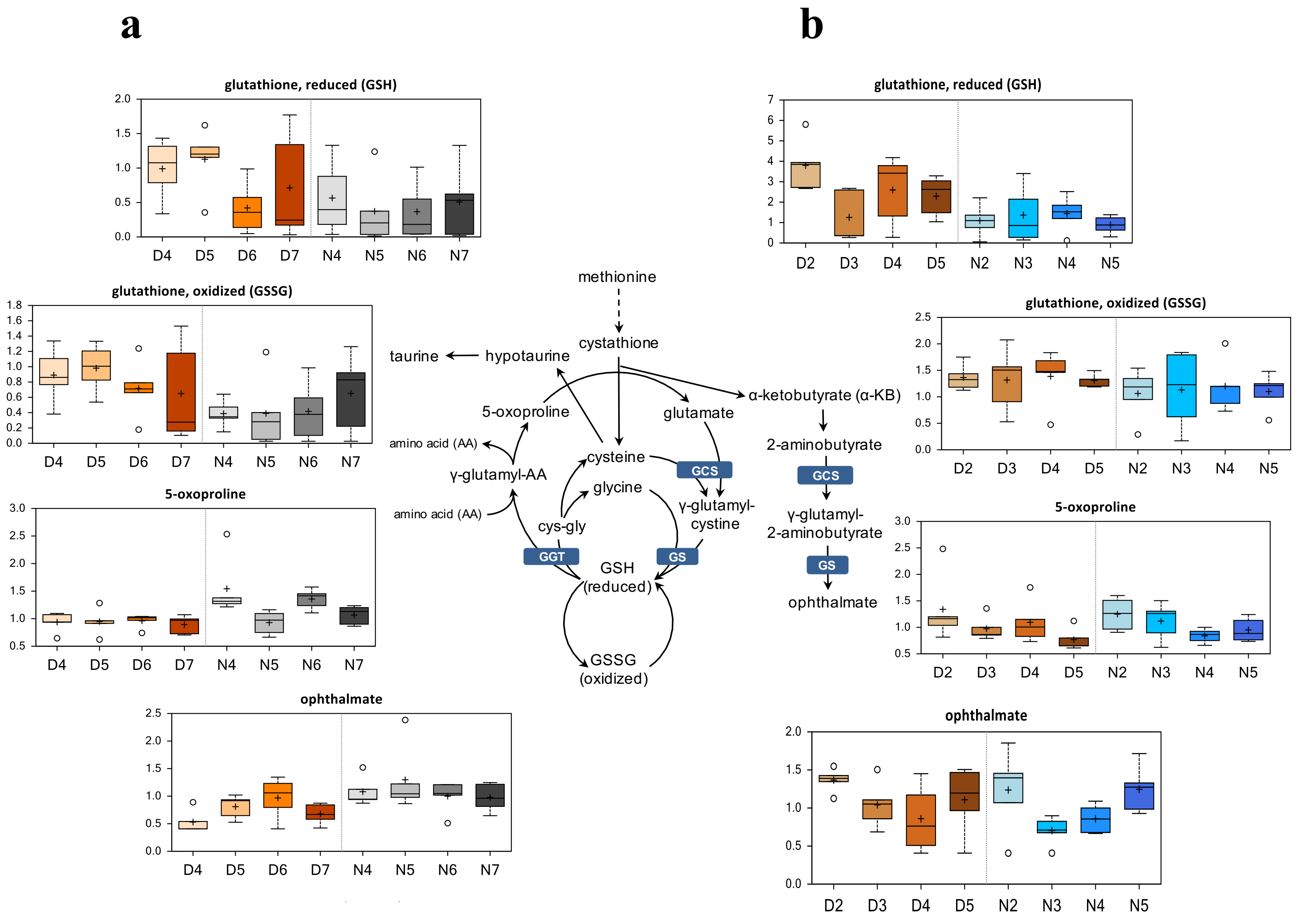

a
b
glutathione, reduced (GSH)
glutathione, oxidized (GSSG)
5-oxoproline
ophthalmate
D4 D5 D6 D7 N4 N5 N6 N7
D2 D3 D4 D5 N2 N3 N4 N5
methionine
cystathione
taurine
hypotaurine
α-ketobutyrate (α-KB)
2-aminobutyrate
GCS
γ-glutamyl-
2-aminobutyrate
GS
ophthalmate
5-oxoproline
glutamate
amino acid (AA)
γ-glutamyl-AA
cysteine
glycine
cys-gly
γ-glutamyl-
cystine
GGT
GSH
(reduced)
GSSG
(oxidized)

Figure S6. Differences in the abundances of metabolites involved in glutathione metabolism in flight muscle of *Manduca sexta* across Diel Time and Age. (**a**) Female, (**b**) Male. The *x*-axis represents age post-eclosion at Day (D) and Night (N). The *y*-axis box plots indicate the scaled intensity mean (+) and median (–) values: upper and lower ranges of boxes indicate upper and lower quartiles, respectively; upper and lower whiskers indicate the maximum and minimum distributions of the data; small circles represent outlying data points. The adjusted *P* values for the significant comparisons are presented in Tables S1 and S2.

Table S1. Metabolite abundances significantly changed in flight muscle of female *Manduca sexta* across Diel Time (adjusted *P* vales) and Age (adjusted *P* vales).

Biochemical	Super Pathway	Sub Pathway	Diel Time	Age	Interaction
carboxyethyl-GABA	Amino Acid	Glutamate Metabolism	0.049799	0.031401	0.76582
glutamine	Amino Acid	Glutamate Metabolism	0.00065014	0.403	0.45242
N-acetylglutamine	Amino Acid	Glutamate Metabolism	0.0010204	0.27076	0.67318
5-oxoproline	Amino Acid	Glutathione Metabolism	0.013372	0.22411	0.42003
ophthalmate	Amino Acid	Glutathione Metabolism	0.01945	0.5067	0.65333
N-acetylglycine	Amino Acid	Glycine, Serine and Threonine Metabolism	0.0024194	0.25474	0.66506
N-acetylserine	Amino Acid	Glycine, Serine and Threonine Metabolism	0.049799	0.51779	0.76675
1-methylhistidine	Amino Acid	Histidine Metabolism	0.6685	0.025967	0.45192
1-methylimidazoleacetate	Amino Acid	Histidine Metabolism	0.81553	0.048871	0.49335
3-methylhistidine	Amino Acid	Histidine Metabolism	0.0007273	0.48717	0.45024
N-acetylhistidine	Amino Acid	Histidine Metabolism	0.0089421	0.84598	0.52691
ethylmalonate	Amino Acid	Leucine, Isoleucine and Valine Metabolism	0.013372	0.67231	0.87193
N-acetylisoleucine	Amino Acid	Leucine, Isoleucine and Valine Metabolism	0.0065931	0.38888	0.6373
N-acetylleucine	Amino Acid	Leucine, Isoleucine and Valine Metabolism	0.0095392	0.33641	0.60154
N-acetylvaline	Amino Acid	Leucine, Isoleucine and Valine Metabolism	0.00098031	0.28677	0.42003
5-(galactosylhydroxy)-L-lysine	Amino Acid	Lysine Metabolism	0.67811	0.011837	0.45242
5-hydroxylysine	Amino Acid	Lysine Metabolism	0.8684	8.1354E-06	0.67766
2-aminobutyrate	Amino Acid	Methionine, Cysteine, SAM and Taurine Metabolism	0.4265	0.031401	0.29311
cysteine sulfinic acid	Amino Acid	Methionine, Cysteine, SAM and Taurine Metabolism	0.027646	0.16323	0.45242
N-formylmethionine	Amino Acid	Methionine, Cysteine, SAM and Taurine Metabolism	0.53727	0.0094609	0.60681
S-adenosylhomocysteine (SAH)	Amino Acid	Methionine, Cysteine, SAM and Taurine Metabolism	0.030129	0.27441	0.80184

S-adenosylmethionine (SAM)	Amino Acid	Methionine, Cysteine, SAM and Taurine Metabolism	0.0075507	8.1354E-06	0.076388
S-methylcysteine sulfoxide	Amino Acid	Methionine, Cysteine, SAM and Taurine Metabolism	0.23814	0.00046907	0.41263
5-methylthioadenosine (MTA)	Amino Acid	Polyamine Metabolism	0.85679	1.7536E-06	0.19957
putrescine	Amino Acid	Polyamine Metabolism	0.00011652	0.41304	0.55405
N-methylproline	Amino Acid	Urea cycle; Arginine and Proline Metabolism	0.77607	0.0059597	0.78083
N-acetyl-glucosamine 1-phosphate	Carbohydrate	Aminosugar Metabolism	0.69306	0.0094609	0.76613
galactonate	Carbohydrate	Fructose, Mannose and Galactose Metabolism	0.0089421	0.20027	0.20365
mannose	Carbohydrate	Fructose, Mannose and Galactose Metabolism	0.037906	0.92288	0.56818
sedoheptulose	Carbohydrate	Pentose Metabolism	0.038608	0.011543	0.14221
alpha-lipoate	Cofactors and Vitamins	Lipoate Metabolism	0.010133	0.68379	0.90538
nicotinate	Cofactors and Vitamins	Nicotinate and Nicotinamide Metabolism	0.17154	0.04186	0.6383
ceramide (d14:1/22:0, d16:1/20:0)*	Lipid	Ceramides	0.39394	0.0089559	0.22261
diacylglycerol (16:1/18:2 [2], 16:0/18:3 [1])*	Lipid	Diacylglycerol	0.00013111	0.2861	0.6373
linoleoyl-arachidonoyl-glycerol (18:2/20:4) [2]*	Lipid	Diacylglycerol	0.028882	0.30026	0.66846
linoleoyl-linolenoyl-glycerol (18:2/18:3) [1]*	Lipid	Diacylglycerol	0.0010421	0.035738	0.98362
linoleoyl-linolenoyl-glycerol (18:2/18:3) [2]*	Lipid	Diacylglycerol	0.00013111	0.30569	0.57876
linoleoyl-linoleoyl-glycerol (18:2/18:2) [1]*	Lipid	Diacylglycerol	0.00031998	0.068536	0.39821
linoleoyl-linoleoyl-glycerol (18:2/18:2) [2]*	Lipid	Diacylglycerol	0.0020905	0.43458	0.62912
myristoyl-linoleoyl-glycerol (14:0/18:2) [1]*	Lipid	Diacylglycerol	0.0013126	0.46307	0.7791
oleoyl-linolenoyl-glycerol (18:1/18:3) [2]*	Lipid	Diacylglycerol	0.00013111	0.19796	0.65333

oleoyl-linoleoyl-glycerol (18:1/18:2) [1]	Lipid	Diacylglycerol	0.011086	0.26347	0.58404
oleoyl-linoleoyl-glycerol (18:1/18:2) [2]	Lipid	Diacylglycerol	0.00054397	0.060875	0.41486
palmitoleoyl-linoleoyl-glycerol (16:1/18:2) [1]*	Lipid	Diacylglycerol	0.00010926	0.18629	0.52691
palmitoyl-linolenoyl-glycerol (16:0/18:3) [2]*	Lipid	Diacylglycerol	0.015094	0.5067	0.70895
palmitoyl-linoleoyl-glycerol (16:0/18:2) [2]*	Lipid	Diacylglycerol	0.0062092	0.020623	0.45242
palmitoyl-oleoyl-glycerol (16:0/18:1) [1]*	Lipid	Diacylglycerol	0.0089421	0.0089559	0.45242
palmitoyl-oleoyl-glycerol (16:0/18:1) [2]*	Lipid	Diacylglycerol	0.011086	0.0047047	0.19957
stearoyl-linoleoyl-glycerol (18:0/18:2) [2]*	Lipid	Diacylglycerol	0.062262	0.0222	0.55156
linoleoylcholine*	Lipid	Fatty Acid Metabolism (Acyl Choline)	0.00013111	0.34827	0.55156
oleoylcholine	Lipid	Fatty Acid Metabolism (Acyl Choline)	0.00045655	0.16317	0.6815
butyrylcarnitine (C4)	Lipid	Fatty Acid Metabolism (also BCAA Metabolism)	0.025806	0.53098	0.66506
3-hydroxybutyrylcarnitine (2)	Lipid	Fatty Acid Metabolism(Acyl Carnitine)	0.0036007	0.0094609	0.60681
acetylcarnitine (C2)	Lipid	Fatty Acid Metabolism(Acyl Carnitine)	0.024941	0.59396	0.076388
arachidoylcarnitine (C20)*	Lipid	Fatty Acid Metabolism(Acyl Carnitine)	0.000087389	0.096351	0.25021
behenoylcarnitine (C22)*	Lipid	Fatty Acid Metabolism(Acyl Carnitine)	0.0063505	0.0056262	0.55156
dihomo-linoleoylcarnitine (C20:2)*	Lipid	Fatty Acid Metabolism(Acyl Carnitine)	0.050663	0.068463	0.0055993
erucoylcarnitine (C22:1)*	Lipid	Fatty Acid Metabolism(Acyl Carnitine)	0.0080152	0.81207	0.78977
oleoylcarnitine (C18:1)	Lipid	Fatty Acid Metabolism(Acyl Carnitine)	0.67539	0.27478	0.021946
stearoylcarnitine (C18)	Lipid	Fatty Acid Metabolism(Acyl Carnitine)	0.73186	0.18332	0.0055993
3,4-methylene heptanoylglycine	Lipid	Fatty Acid Metabolism(Acyl Glycine)	0.00050989	0.49738	0.6815
hexanoylglycine	Lipid	Fatty Acid Metabolism(Acyl Glycine)	0.037906	0.6125	0.71186
N-linoleoylglycine	Lipid	Fatty Acid Metabolism(Acyl Glycine)	0.047741	0.33641	0.60154
2-aminoheptanoate	Lipid	Fatty Acid, Amino	0.73423	0.0054704	0.68933
2-aminooctanoate	Lipid	Fatty Acid, Amino	0.77607	0.0051259	0.9313

3-hydroxypropanoate	Lipid	Fatty Acid, Monohydroxy	0.014406	0.33641	0.80534
3-hydroxysebacate	Lipid	Fatty Acid, Monohydroxy	0.00090246	0.41304	0.58404
galactosylglycerol*	Lipid	Glycerolipid Metabolism	0.70817	0.0015198	0.76958
glycerol	Lipid	Glycerolipid Metabolism	0.000052173	0.36343	0.58271
linolenoyl-linolenoyl-glycerol (18:3/18:3) [1]*	Lipid	Glycolipid Metabolism	0.0024194	0.36146	0.66425
linolenoyl-linolenoyl-glycerol (18:3/18:3) [2]*	Lipid	Glycolipid Metabolism	0.0022964	0.84373	0.99631
3-hydroxybutyrate (BHBA)	Lipid	Ketone Bodies	0.0066988	0.40513	0.85776
10-heptadecenoate (17:1n7)	Lipid	Long Chain Fatty Acid	0.02113	0.54578	0.81072
10-nonadecenoate (19:1n9)	Lipid	Long Chain Fatty Acid	0.049189	0.33745	0.6373
arachidate (20:0)	Lipid	Long Chain Fatty Acid	0.0065931	0.46168	0.88032
eicosenoate (20:1)	Lipid	Long Chain Fatty Acid	0.028384	0.26373	0.55621
palmitate (16:0)	Lipid	Long Chain Fatty Acid	0.028204	0.36478	0.72514
palmitoleate (16:1n7)	Lipid	Long Chain Fatty Acid	0.01997	0.65493	0.72514
1-linolenoyl-GPC (18:3)*	Lipid	Lysolipid	0.0036007	0.16818	0.6815
1-linoleoyl-GPE (18:2)*	Lipid	Lysolipid	0.0062092	0.1279	0.71141
1-linoleoyl-GPI (18:2)*	Lipid	Lysolipid	0.021535	0.30823	0.47788
1-oleoyl-GPE (18:1)	Lipid	Lysolipid	0.002019	0.013057	0.41099
1-oleoyl-GPG (18:1)*	Lipid	Lysolipid	0.028204	0.22996	0.82618
1-oleoyl-GPI (18:1)*	Lipid	Lysolipid	0.049799	0.28677	0.55036
1-palmitoleoyl-GPC (16:1)*	Lipid	Lysolipid	0.022354	0.22248	0.71186
1-palmitoyl-GPC (16:0)	Lipid	Lysolipid	0.013372	0.22996	0.67766
1-palmitoyl-GPE (16:0)	Lipid	Lysolipid	0.0039348	0.0015198	0.38213
1-stearoyl-GPC (18:0)	Lipid	Lysolipid	0.045363	0.052966	0.67318
1-stearoyl-GPE (18:0)	Lipid	Lysolipid	0.003327	0.00033463	0.5416
2-palmitoleoyl-GPC (16:1)*	Lipid	Lysolipid	0.028384	0.53687	0.6373
1-(1-enyl-oleoyl)-2-linoleoyl-GPE (P-18:1/18:2)*	Lipid	Lysoplasmalogen	0.003202	0.40513	0.20365
1-(1-enyl-oleoyl)-GPE (P-18:1)*	Lipid	Lysoplasmalogen	0.020868	0.010128	0.54069
1-(1-enyl-palmitoyl)-GPE (P-16:0)*	Lipid	Lysoplasmalogen	0.010031	0.0222	0.5744

1-(1-enyl-stearoyl)-GPE (P-18:0)*	Lipid	Lysoplasmalogen	0.0036007	0.0059597	0.43668
1-linoleoylglycerol (18:2)	Lipid	Monoacylglycerol	0.02164	0.70118	0.6815
1-oleoyl-2-linoleoyl-GPE (18:1/18:2)*	Lipid	Phospholipid Metabolism	0.032064	0.27218	0.986
1-stearoyl-2-linoleoyl-GPE (18:0/18:2)*	Lipid	Phospholipid Metabolism	0.00327	0.79543	0.66337
1-stearoyl-2-oleoyl-GPE (18:0/18:1)	Lipid	Phospholipid Metabolism	0.012501	0.70847	0.89084
1-stearoyl-2-oleoyl-GPI (18:0/18:1)*	Lipid	Phospholipid Metabolism	0.049799	0.84598	0.93714
choline	Lipid	Phospholipid Metabolism	0.0039348	0.92748	0.41099
choline phosphate	Lipid	Phospholipid Metabolism	0.0080152	0.5746	0.42003
ethanolamine	Lipid	Phospholipid Metabolism	0.0024194	0.017032	0.53721
glycerophosphoethanolamine	Lipid	Phospholipid Metabolism	0.040474	0.39548	0.74534
glycerophosphorylcholine (GPC)	Lipid	Phospholipid Metabolism	0.030129	0.87636	0.60154
1-(1-enyl-stearoyl)-2-linoleoyl-GPE (P-18:0/18:2)*	Lipid	Plasmalogen	0.049799	0.33723	0.66425
dihomo-linoleate (20:2n6)	Lipid	Polyunsaturated Fatty Acid (n3 and n6)	0.038608	0.26373	0.60154
dihomo-linolenate (20:3n3 or n6)	Lipid	Polyunsaturated Fatty Acid (n3 and n6)	0.0094963	0.28318	0.75603
docosadienoate (22:2n6)	Lipid	Polyunsaturated Fatty Acid (n3 and n6)	0.019197	0.32337	0.5564
linoleate (18:2n6)	Lipid	Polyunsaturated Fatty Acid (n3 and n6)	0.019472	0.59396	0.90772
linolenate [alpha or gamma; (18:3n3 or 6)]	Lipid	Polyunsaturated Fatty Acid (n3 and n6)	0.019785	0.55271	0.90201
ceramide (d14:1/18:0, d16:1/16:0)*	Lipid	Sphingolipid Metabolism	0.0026946	0.029911	0.29311
myristoyl dihydrosphingomyelin (d18:0/14:0)*	Lipid	Sphingolipid Metabolism	0.017255	0.1571	0.65976
N-palmitoyl-sphinganine (d18:0/16:0)	Lipid	Sphingolipid Metabolism	0.00090246	0.49937	0.88059
palmitoyl dihydrosphingomyelin (d18:0/16:0)*	Lipid	Sphingolipid Metabolism	0.028204	0.48717	0.38068
palmitoyl sphingomyelin (d18:1/16:0)	Lipid	Sphingolipid Metabolism	0.00041492	0.32924	0.58489
sphingomyelin (d18:1/14:0, d16:1/16:0)*	Lipid	Sphingolipid Metabolism	0.027646	0.30635	0.42553
sphingomyelin (d18:1/15:0, d16:1/17:0)*	Lipid	Sphingolipid Metabolism	0.049799	0.46168	0.91035
sphingomyelin (d18:1/18:1, d18:2/18:0)	Lipid	Sphingolipid Metabolism	0.035327	0.65986	0.72636

sphingomyelin (d18:2/14:0, d18:1/14:1)*	Lipid	Sphingolipid Metabolism	0.024106	0.25877	0.66425
sphingomyelin (d18:2/16:0, d18:1/16:1)*	Lipid	Sphingolipid Metabolism	0.019472	0.18629	0.67974
tetradecanoylsphingosine (d14:1)*	Lipid	Sphingolipid Metabolism	0.96168	0.031401	0.71186
1-methyladenine	Nucleotide	Purine Metabolism, Adenine containing	0.99161	0.031401	0.42003
N1-methyladenosine	Nucleotide	Purine Metabolism, Adenine containing	0.00013111	0.080883	0.41099
guanosine	Nucleotide	Purine Metabolism, Guanine containing	0.027646	0.14582	0.60146
3-methylcytidine	Nucleotide	Pyrimidine Metabolism, Cytidine containing	0.62496	0.0051996	0.55156
pseudouridine	Nucleotide	Pyrimidine Metabolism, Uracil containing	0.093038	0.0054704	0.66425
uridine 3'-monophosphate (3'-UMP)	Nucleotide	Pyrimidine Metabolism, Uracil containing	0.017279	0.3653	0.99115
glycylleucine	Peptide	Dipeptide	0.35586	0.0222	0.71505
histidylalanine	Peptide	Dipeptide	0.89793	0.020615	0.80534
isoleucylglycine	Peptide	Dipeptide	0.56469	0.013593	0.76958
leucylglycine	Peptide	Dipeptide	0.70141	0.010128	0.65333
lysylleucine	Peptide	Dipeptide	0.92178	0.0094609	0.33715
phenylalanylalanine	Peptide	Dipeptide	0.53706	0.0076268	0.45242
phenylalanylglycine	Peptide	Dipeptide	0.45586	0.013057	0.55405
tryptophylglycine	Peptide	Dipeptide	0.99125	0.0056262	0.52419
valylglycine	Peptide	Dipeptide	0.55267	0.0059535	0.74319
valylleucine	Peptide	Dipeptide	0.91278	0.014055	0.39821
gamma-glutamylglutamine	Peptide	Gamma-glutamyl Amino Acid	0.026578	0.86435	0.6815
4-hydroxybenzoate	Xenobiotics	Benzoate Metabolism	0.043607	0.25474	0.41099
4-hydroxyhippurate	Xenobiotics	Benzoate Metabolism	0.0022761	8.1354E-06	6.5492E-06
methyl-4-hydroxybenzoate sulfate	Xenobiotics	Benzoate Metabolism	0.014375	0.014291	0.057052
ectoine	Xenobiotics	Chemical	0.77607	0.010128	0.87177

Table S2. Metabolite abundances significantly changed in flight muscle of male *Manduca sexta* across Diel Time (adjusted *P* vales) and Age (adjusted *P* vales).

Biochemical	Super Pathway	Sub Pathway	Diel Time	Age	Interaction
cyano-alanine	Amino Acid	Alanine and Aspartate Metabolism	0.70472	0.029397	0.67405
carboxyethyl-GABA	Amino Acid	Glutamate Metabolism	0.69577	0.013066	0.74498
glutamate, gamma-methyl ester	Amino Acid	Glutamate Metabolism	0.86709	0.013066	0.86846
glutamine	Amino Acid	Glutamate Metabolism	0.7922	0.013066	0.63066
glutathione, reduced (GSH)	Amino Acid	Glutathione Metabolism	0.045249	0.31154	0.39955
glycine	Amino Acid	Glycine, Serine and Threonine Metabolism	0.027229	0.0001396	0.38885
phosphothreonine	Amino Acid	Glycine, Serine and Threonine Metabolism	0.53599	0.0011741	0.38885
threonine	Amino Acid	Glycine, Serine and Threonine Metabolism	0.57931	0.022578	0.34782
5-hydroxylysine	Amino Acid	Lysine Metabolism	0.85049	0.000008979	0.85004
S-adenosylhomocysteine (SAH)	Amino Acid	Methionine, Cysteine, SAM and Taurine Metabolism	0.5573	0.004749	0.36303
S-adenosylmethionine (SAM)	Amino Acid	Methionine, Cysteine, SAM and Taurine Metabolism	0.2506	4.2848E-06	0.88569
S-methylcysteine sulfoxide	Amino Acid	Methionine, Cysteine, SAM and Taurine Metabolism	0.87746	0.030348	0.69612
5-methylthioadenosine (MTA)	Amino Acid	Polyamine Metabolism	0.0041035	0.00021528	0.31674
N-alpha-acetylornithine	Amino Acid	Urea cycle; Arginine and Proline Metabolism	0.44561	0.020832	0.69612
N-methylproline	Amino Acid	Urea cycle; Arginine and Proline Metabolism	0.18741	0.013066	0.5613
N-acetyl-glucosamine 1-phosphate	Carbohydrate	Aminosugar Metabolism	0.94171	0.0024711	0.83192
sedoheptulose	Carbohydrate	Pentose Metabolism	0.69984	0.0090891	0.57844
biotin	Cofactors and Vitamins	Biotin Metabolism	0.70472	2.1364E-06	0.17815
alpha-lipoate	Cofactors and Vitamins	Lipoate Metabolism	0.85049	0.0048537	0.83192
phosphopantetheine	Cofactors and Vitamins	Pantothenate and CoA Metabolism	0.019195	0.20426	0.42504

xanthopterin	Cofactors and Vitamins	Pterin Metabolism	0.69573	0.020832	0.71957
thiamin (Vitamin B1)	Cofactors and Vitamins	Thiamine Metabolism	0.47247	0.040757	0.52397
pyridoxal	Cofactors and Vitamins	Vitamin B6 Metabolism	0.18741	0.020832	0.8909
pyridoxamine	Cofactors and Vitamins	Vitamin B6 Metabolism	0.026304	0.075985	0.82458
aconitate [cis or trans]	Energy	TCA Cycle	0.027229	0.14597	0.40193
citrate	Energy	TCA Cycle	0.0072479	0.33152	0.47651
isocitrate	Energy	TCA Cycle	0.010198	0.3903	0.57063
ceramide (d14:1/22:0, d16:1/20:0)*	Lipid	Ceramides	0.48808	2.1623E-07	0.8481
ceramide (d18:1/20:0, d16:1/22:0, d20:1/18:0)*	Lipid	Ceramides	0.9358	0.00061658	0.8481
oleoyl ethanolamide	Lipid	Endocannabinoid	0.42715	0.004271	0.4137
butyrylcarnitine (C4)	Lipid	Fatty Acid Metabolism (also BCAA Metabolism)	0.0041035	0.004271	0.14278
3-hydroxybutyrylcarnitine (1)	Lipid	Fatty Acid Metabolism(Acyl Carnitine)	0.49718	0.01547	0.57844
3-hydroxybutyrylcarnitine (2)	Lipid	Fatty Acid Metabolism(Acyl Carnitine)	0.20096	0.0033479	0.6395
acetylcarnitine (C2)	Lipid	Fatty Acid Metabolism(Acyl Carnitine)	0.48599	0.033991	0.31674
lignoceroylcarnitine (C24)*	Lipid	Fatty Acid Metabolism(Acyl Carnitine)	0.95497	0.024596	0.82458
N-linoleoylglycine	Lipid	Fatty Acid Metabolism(Acyl Glycine)	0.57931	0.0018523	0.76287
2-aminoheptanoate	Lipid	Fatty Acid, Amino	0.30542	0.000050153	0.38885
2-aminooctanoate	Lipid	Fatty Acid, Amino	0.59286	0.043964	0.99319
2-hydroxyglutarate	Lipid	Fatty Acid, Dicarboxylate	0.90944	0.0033647	0.75731
3-hydroxydecanoate	Lipid	Fatty Acid, Monohydroxy	0.53599	0.025603	0.69612
3-hydroxylaurate	Lipid	Fatty Acid, Monohydroxy	0.98012	0.034914	0.80904
3-hydroxysebacate	Lipid	Fatty Acid, Monohydroxy	0.49498	0.0064107	0.14278
galactosylglycerol*	Lipid	Glycerolipid Metabolism	0.0095563	7.9612E-10	0.77924
glycerol	Lipid	Glycerolipid Metabolism	0.026304	0.025872	0.14278

3-hydroxybutyrate (BHBA)	Lipid	Ketone Bodies	0.60843	0.020832	0.57844
behenate (22:0)*	Lipid	Long Chain Fatty Acid	0.93222	0.0058157	0.66726
margarate (17:0)	Lipid	Long Chain Fatty Acid	0.81802	0.0049508	0.86686
oleate/vaccenate (18:1)	Lipid	Long Chain Fatty Acid	0.62332	0.044882	0.75486
palmitate (16:0)	Lipid	Long Chain Fatty Acid	0.775	0.011468	0.80076
stearate (18:0)	Lipid	Long Chain Fatty Acid	0.70275	0.04773	0.75486
1-linolenoyl-GPC (18:3)*	Lipid	Lysolipid	0.77998	0.020832	0.44489
1-linoleoyl-GPE (18:2)*	Lipid	Lysolipid	0.66821	0.044914	0.56582
1-palmitoyl-GPC (16:0)	Lipid	Lysolipid	0.53918	0.00017892	0.36303
1-palmitoyl-GPE (16:0)	Lipid	Lysolipid	0.64365	0.00010755	0.378
1-palmitoyl-GPG (16:0)*	Lipid	Lysolipid	0.98012	0.0018862	0.36303
1-stearoyl-GPE (18:0)	Lipid	Lysolipid	0.70046	0.025603	0.40449
2-palmitoleoyl-GPC (16:1)*	Lipid	Lysolipid	0.70472	0.0075848	0.77109
2-palmitoyl-GPC (16:0)*	Lipid	Lysolipid	0.98037	0.0019504	0.378
1-(1-enyl-oleoyl)-GPE (P-18:1)*	Lipid	Lysoplasmalogen	0.80133	0.01315	0.36303
1-stearoyl-2-oleoyl-GPS (18:0/18:1)	Lipid	Phosphatidylserine (PS)	0.7066	0.004271	0.31674
1-palmitoyl-2-linolenoyl-GPG (16:0/18:3)*	Lipid	Phospholipid Metabolism	0.016755	0.0095203	0.54553
1-palmitoyl-2-linoleoyl-GPC (16:0/18:2)	Lipid	Phospholipid Metabolism	0.2436	0.0041445	0.42504
1-palmitoyl-2-linoleoyl-GPE (16:0/18:2)	Lipid	Phospholipid Metabolism	0.15233	3.4916E-07	0.378
1-palmitoyl-2-linoleoyl-GPG (16:0/18:2)	Lipid	Phospholipid Metabolism	0.049153	0.96333	0.60866
1-palmitoyl-2-oleoyl-GPC (16:0/18:1)	Lipid	Phospholipid Metabolism	0.13778	0.00060588	0.31674
1-palmitoyl-2-oleoyl-GPE (16:0/18:1)	Lipid	Phospholipid Metabolism	0.13778	0.000008979	0.34297
1-stearoyl-2-arachidonoyl-GPE (18:0/20:4)	Lipid	Phospholipid Metabolism	0.5573	0.013066	0.81041
choline phosphate	Lipid	Phospholipid Metabolism	0.95497	0.0041808	0.14278
docosadienoate (22:2n6)	Lipid	Polyunsaturated Fatty Acid (n3 and n6)	0.62332	0.020832	0.31674
linoleate (18:2n6)	Lipid	Polyunsaturated Fatty Acid (n3 and n6)	0.70472	0.04773	0.57844

linolenate [alpha or gamma; (18:3n3 or 6)]	Lipid	Polyunsaturated Fatty Acid (n3 and n6)	0.65445	0.020832	0.57063
ceramide (d14:1/18:0, d16:1/16:0)*	Lipid	Sphingolipid Metabolism	0.27788	0.0075848	0.56582
hexadecasphinganine (d16:0)*	Lipid	Sphingolipid Metabolism	0.63574	0.019366	0.66159
N-arachidoyl-tetradecanoylsphingosine (d14:1/20:0)*	Lipid	Sphingolipid Metabolism	0.93297	0.0075375	0.95785
N-behenoyl-tetradecanoylsphinganine (d14:0/22:0)*	Lipid	Sphingolipid Metabolism	0.84515	0.024793	0.49189
N-stearoyl-hexadecasphingosine (d17:1/18:0)*	Lipid	Sphingolipid Metabolism	0.17735	0.00014716	0.40035
tetradecasphinganine (d14:0)*	Lipid	Sphingolipid Metabolism	0.026304	0.013066	0.40035
methylphosphate	Nucleotide	Purine and Pyrimidine Metabolism	0.037617	0.049034	0.42504
inosine 5'-monophosphate (IMP)	Nucleotide	Purine Metabolism, (Hypo)Xanthine/Inosine containing	0.30587	0.00079954	0.14278
N1-methylinosine	Nucleotide	Purine Metabolism, (Hypo)Xanthine/Inosine containing	0.5573	0.030348	0.72223
2'-deoxyadenosine	Nucleotide	Purine Metabolism, Adenine containing	0.22802	0.00064937	0.49189
adenosine	Nucleotide	Purine Metabolism, Adenine containing	0.65445	0.00064937	0.57844
N1-methyladenosine	Nucleotide	Purine Metabolism, Adenine containing	0.049153	0.000058595	0.36303
guanosine	Nucleotide	Purine Metabolism, Guanine containing	0.18741	0.000026902	0.18462
guanosine 5'- monophosphate (5'-GMP)	Nucleotide	Purine Metabolism, Guanine containing	0.015708	0.075985	0.14278
3-methylcytidine	Nucleotide	Pyrimidine Metabolism, Cytidine containing	0.23601	0.00098419	0.14278
3-aminoisobutyrate	Nucleotide	Pyrimidine Metabolism, Thymine containing	0.24017	3.3411E-07	0.57844
5-methyluridine (ribothymidine)	Nucleotide	Pyrimidine Metabolism, Uracil containing	0.93222	0.0033647	0.77109
beta-alanine	Nucleotide	Pyrimidine Metabolism, Uracil containing	0.84212	0.00014716	0.69612
N3-methyluridine	Nucleotide	Pyrimidine Metabolism, Uracil containing	0.69984	0.0064454	0.73469

pseudouridine	Nucleotide	Pyrimidine Metabolism, Uracil containing	0.94757	5.8399E-06	0.85202
uridine 3'-monophosphate (3'-UMP)	Nucleotide	Pyrimidine Metabolism, Uracil containing	0.30542	0.000012431	0.57844
4-hydroxyhippurate	Xenobiotics	Benzoate Metabolism	0.35482	0.034197	0.34782
